# Carbohydrate Availability Regulates Virulence Gene Expression in *Streptococcus suis*

**DOI:** 10.1371/journal.pone.0089334

**Published:** 2014-03-18

**Authors:** M. Laura Ferrando, Peter van Baarlen, Germano Orrù, Rosaria Piga, Roger S. Bongers, Michiel Wels, Astrid De Greeff, Hilde E. Smith, Jerry M. Wells

**Affiliations:** 1 Host-Microbe Interactomics, Animal Sciences, Wageningen University, Wageningen, The Netherlands; 2 Department of Medical Microbiology, Academic Medical Center, Amsterdam, The Netherlands; 3 Oral Biotechnology Laboratory, University of Cagliari, Cagliari, Italy; 4 NIZO Food Research B.V., Ede, The Netherlands; 5 Central Veterinary Institute, Animal Sciences Group, Wageningen University, Lelystad, The Netherlands; University of Oklahoma Health Sciences Center, United States of America

## Abstract

*Streptococcus suis* is a major bacterial pathogen of young pigs causing worldwide economic problems for the pig industry. *S. suis* is also an emerging pathogen of humans. Colonization of porcine oropharynx by *S. suis* is considered to be a high risk factor for invasive disease. In the oropharyngeal cavity, where glucose is rapidly absorbed but dietary α-glucans persist, there is a profound effect of carbohydrate availability on the expression of virulence genes. Nineteen predicted or confirmed *S. suis* virulence genes that promote adhesion to and invasion of epithelial cells were expressed at higher levels when *S. suis* was supplied with the α-glucan starch/pullulan compared to glucose as the single carbon source. Additionally the production of suilysin, a toxin that damages epithelial cells, was increased more than ten-fold when glucose levels were low and *S. suis* was growing on pullulan. Based on biochemical, bioinformatics and *in vitro* and *in vivo* gene expression studies, we developed a biological model that postulates the effect of carbon catabolite repression on expression of virulence genes in the mucosa, organs and blood. This research increases our understanding of *S. suis* virulence mechanisms and has important implications for the design of future control strategies including the development of anti-infective strategies by modulating animal feed composition.

## Introduction

*Streptococcus suis* is a major bacterial pathogen of young pigs and a worldwide economic problem for the pig industry. Furthermore, *S. suis* is emerging as a zoonotic pathogen associated with meningitis and septicaemia in humans [1], [2]. In pigs, invasive disease is thought to be caused by translocation of *S. suis* across the mucosal epithelium in the upper respiratory tract [3], [4]. The ecological conditions that promote adhesion to, and invasion of the host mucosa by *S. suis* are unknown and probably depend on the environmental conditions and the bacterial genotype including presence of virulence genes. *In vitro* studies on adhesion and invasion by *S. suis* have often been performed in medium containing glucose as a carbon source which does not accurately reflect the situation *in vivo*. In the oropharyngeal cavity including the saliva, glucose may be present but concentrations usually diminish rapidly (within 30 min) after ingestion [5], [6] as glucose is readily absorbed by the host and metabolized by commensal bacteria. In contrast, starch α-glucans, large polymers of D-glucose that are present in large amounts in animal feeds [7] can persist in high concentrations in the oropharynx of humans and pigs [8]–[11]. A second type of carbohydrate that may promote proliferation of pathogenic bacteria is animal glycogen released from damaged or lysed cells. Suilysin, a hemolytic toxin encoded by the *S. suis sly* gene, may release, through its cytotoxic effect on epithelial cells, cellular glycogen [12], [13] that may serve as an important substrate for pathogen growth during the early stages of infection.

Currently, little is known about the expression and regulation of *S. suis* virulence factors such as suilysin. Streptococcal pathogens contain genes required for efficient utilization of α-glucans including amylases and/or pullulanases which cleave α-1,4 and α-1,6 glycosidic bonds in starch or glycogen [14]–[18]. Previously, we have shown that a cell wall anchored amylopullulanase of *S. suis* serotype 2 (*apuA*-SSU1849) was necessary to support bacterial proliferation on the α-glucan starch/pullulan (an α-1,6; α-1,4 linked glucose polymer) as a single carbon source and to promote adhesion of *S. suis* to porcine tracheal epithelial cells [19].

As in all Gram-positive bacteria, in *S. suis* the expression of carbohydrate metabolic enzymes are under the control of the global transcriptional regulator, catabolite control protein A (CcpA) that mediates carbon catabolite control (CCC) in presence of a preferred sugar, usually glucose. CcpA can repress or activate transcription by binding to *cis*-acting catabolite response element (*cre*) sites in the gene promoter regions [20], [21].

Given the relative abundance of dietary complex carbohydrates such as α-glucans and low level of glucose in the porcine upper oropharyngeal niche occupied by *S. suis*, we compared the genome-wide effects of growth on glucose or the α-glucan starch/pullulan on *S. suis* metabolism and virulence gene expression. We first constructed a metabolic map for *S. suis* and used it to analyze the key metabolic pathways altered by growth on glucose or pullulan. In addition to genes that play roles in (carbohydrate) metabolic pathways, 19 virulence genes were differentially expressed, seven of which were strongly induced (ratios>10) by growth in pullulan compared to glucose and nine were shown to contain a consensus *cre* site in their promoter sequences. The regulation of *apuA*, the virulence factor most strongly induced in pullulan compared to glucose, was investigated in detail using qPCR analysis of gene transcripts in bacteria grown in different carbon sources, promoter mapping and binding studies with CcpA and a newly identified transcriptional regulator, ApuR. The biological consequences of carbohydrate metabolism and virulence gene expression were also assessed in an *in vitro* porcine tracheal cell model using qPCR, hemolytic assays and adhesion and invasion assays. Based on these findings we propose a model for the transcriptional regulation of production of virulence factors during different stages of infection dependent on a CcpA-mediated, carbon catabolite control-dependent mechanism. To verify the predictions of this model, the *in vivo* expression of *apuA* and *sly* was measured for *S. suis* serotype 2 recovered from the blood and organs of pigs infected under controlled conditions.

## Materials and Methods

### Bacterial strains, plasmids and culture conditions

The virulent *S. suis* serotype 2 strain S10 [22] and S735-pCOM1-V10 [23] were used in this study. The genome of *S. suis* S10 is more than 99% identical to the genome of *S. suis* 2 strain P1/7, a sequenced reference strain of which the genome had been annotated previously (NCBI Genome and NCBI BioProject http://www.ncbi.nlm.nih.gov/genome/?term=Streptococcus%20suis) [24]. *S. suis* was grown in Todd-Hewitt broth (THB) (Difco) or on Columbia agar plates with 6% sheep blood (Oxoid) at 37°C under 5% CO_2_ for 18 hr. A complex medium (CM) (Text S1) was prepared as previously described [19], [25]. The carbohydrates were added separately and sterilized by autoclaving at 100°C for 10 min (pullulan) or filtration with 0.45 µM pore size filter (glucose, lactose and maltotriose). We previously demonstrated that *S. suis* only grows to high density in CM when exogenous carbohydrates are added [19]. Growth in complex medium was determined by measurement of turbidity at OD_600_ using a SpectraMax M5 reader (Molecular Devices LLC).

### RNA extraction from *in vitro* grown *S. suis* and quantitative PCR (qPCR)

For RNA extraction, *S. suis* S10 was grown to exponential (e) and early stationary (s) phase as indicated in Figure S1. Ten ml of culture was collected and centrifugated for each time point. The pellet was immediately frozen in liquid nitrogen until further handling. The frozen pellet was dissolved in 600 µl RA1 reagent (Macherey-Nagel) plus β-mercaptoethanol and lysed using a FastPrep-24 (MP. Biomedicals, Solon, OH) for 6.0 m/sec at 20 sec. Total RNA was purified using NucleoSpin RNA II (Macherey-Nagel). The quality and the concentration of RNA were assessed with an Experion System (Bio-Rad) and by analysis of the A_260_/A_280_ ratio (NanoDrop 8000 UV-Vis Spectrophotometer). For qPCR, cDNA was synthesized using SuperScript VILO (Invitrogen). Primers were designed using Oligo Program version 6 (MedProbe, Oslo, Norway) (Table S1). Quantitative PCR was performed using a LightCycler 4.0 V and the LightCycler FastStart DNA Master SYBR Green I Kit (Roche). Constitutive gene expression in complex media was determined as a ratio of target gene *vs* reference gene *proS* (Text S1) [26]. The level of *proS* expression was constant at all the time points analyzed (data not shown). Two replicates of all samples and primer pairs were included and the experiment was performed in triplicate. Non-template controls were included for each gene in each run.

### Microarray transcriptome analysis

An *S. suis* oligoarray (8×15 K) containing *in situ* synthesized 60-mers was produced by Agilent Technologies (Santa Clara, USA), based on the genome sequence of *S. suis* P1/7 [24]. A total of 7651 unique 60-mers having a theoretical melting temperature of approximately 81°C and representing 1960 ORFs were selected as described [27]. Genes were represented by 4 (91%), 3 (4%), 2 (2%), or 1 probe (3%). Twenty-five putative genes were not represented on the array because no unique probe satisfying the selection criteria could be selected. RNA (1 µg) from *S. suis* samples was labeled using the Cyanine 5 (Cy5) labeling reaction (Amersham Biosciences, Buckinghamshire, UK). Co-hybridization with labeled cDNA probes was performed on these oligonucleotide arrays at 42°C for 16 h in Slidehyb#1 (Ambion, Austin, USA). The data were normalized using Lowess normalization [28] as available in MicroPrep [29] and corrected for inter-slide differences on the basis of total signal intensity per slide using Postprep [29]. Significance of differential gene expression was based on FDR values lower than 0.05. All microarray data are MIAME compliant and available in the NCBI GEO database (http://www.ncbi.nlm.nih.gov/geo/) under accession number GSE40658. Details of the custom-made 60-mer oligonucleotide array design (Agilent Biotechnologies, Amstelveen, The Netherlands) are deposited in the ArrayExpress database (http://www.ebi.ac.uk/arrayexpress/) under accession number A-MEXP-1671.

### Bioinformatic tools: microarray analysis and *cre* motifs search

Differential gene expression of *S. suis* bacteria grown in CM supplemented with pullulan (Pul) or glucose (Glc) and harvested at early exponential (e) or early stationary (s) phase was cross-compared in different combinations (Pul_e vs Glc_s, Pul_s vs Glc_s, Pul_e vs Pul_s and Glc_e vs Glc_s). Overlapping and unique differentials were visualized using Venn diagrams at http://bioinfogp.cnb.csic.es/tools/venny/index.html.

For all genes and proteins identified in the *S. suis* P1/7 genome, Gene Ontology (GO, http://www.geneontology.org) and KEGG pathway annotations were obtained using the BLAST2GO software (www.blast2go.org) [30] including annotations based on terms obtained from EBI using the InterPROScan feature [31] that is part of BLAST2GO.

BLAST2GO was used to annotate all known *S. suis* genes and proteins according to standard Gene Ontology (GO) (www.geneontology.org) nomenclature. BLAST2GO uses the integrated Gossip package [30] for statistical assessment of differences in GO term abundance between two sets of sequences. The GO enrichment analysis feature of BLAST2GO was then used to identify functional GO terms that were statistically over- or underrepresented in the set of genes differentially expressed in pullulan vs glucose in exponential and early stationary phases of growth (Fisher's Exact Test p<0.05).

The MEME (http://meme.sdsc.edu/meme/meme.html) software suite (version 4.1.0) was used for the identification of motifs OM1 and OM2 [32]. As input, we used the promoter sequences of the *MdxE* and *MalE* genes, with well-characterized *cre* sites, from *Bacillus* and *Listeria* (TGWAARCGYTWNCW [W = A or T; R = A or G; Y = C or T; N = any base]). A range of motif widths (15 nt in length) and zero or one motif per sequence were specified in our queries and FIMO, part of MEME, was used to search for this motif in the genome of *S. suis* P1/7. The algorithm in MAST, also part of the MEME suite, calculates position scores for the motif at each possible position within a sequence [33]; motif hits with a position-specific goodness-of-fit *P* value below 10^−4^ were considered to identify putative CcpA binding sites. *In silico* searches and comparisons of predicted *cre* sites within the *S. suis* P1/7 genome sequence and reconstruction of *cre* locations in the predicted operons were conducted using the corresponding databases provided by the MicrobesOnline database web server (http://microbesonline.org) and RegPrecise database [34].

### 5′-Rapid Amplification of cDNA Ends (5′-RACE)

The 5′-rapid amplification of cDNA ends (RACE) system (Invitrogen) was used to determine the transcription start site of the *apuA* gene. Briefly, the first strand cDNA was reverse transcribed from RNA from 1 µg of total RNA *S. suis* grown in CM plus pullulan using the specific primer ASP1 (Table S1). A homopolymeric tail was added to the 3′-end of the cDNA using terminal deoxynucleotidyl transferase (TdT) and the deoxynucleotide dCTP. The tailed cDNA was amplified in nested-PCR with Abridged Anchor Primer (AAP) and a second *apuA* specific primer ASP2 primer upstream ASP1. The resulting 5′-RACE product of ∼380 bp was sequenced and analysed by Vector NTI software (Invitrogen).

### Recombinant regulators production and infrared EMSA

The genes encoding the regulators ApuR (amino acids 2 to 312) and CcpA (amino acids 2 to 333) were amplified by PCR from *S. suis* 2 S10 genomic DNA using GoTaq (Promega) with primers ApuR_F/R and CcpA_F/R respectively (Table S1). The recombinant ApuR (rApuR) and CcpA (rCcpA) proteins were cloned in pTrcHis TOPO2 TA and purified by HPLC affinity chromatography (HisTrap affinity column, Amersham Pharmacia Biotech) as previously described (Text S1) [19]. Fractions containing purified fusion proteins of the expected size (approx. 38 kDa for ApuR and 40 kDa for CcpA) were collected and dialyzed against buffer (500 mM NaCl, 50 mM Tris-HCl, pH 7.4) and stored at −80°C with 10% of glycerol. Protein concentrations were measured using a BCA Protein Assay kit (Thermo Scientific). Further details can be found in the Text S1.

Infrared electrophoretic mobility shift assays (EMSA) were performed utilizing three pairs of fluorescent InfraRed-dye 800 (IRdye 800) labelled primer on the 5′ ends (Biolegio, The Netherlands). The IRdye-primers used to PCR-amplify three DNA fragments of ca. 120 bp (Pr1-Pr3) contained overlapping regions to cover the full length of the *S. suis* P *_apuA_* promoter sequence (Table S1). The IRdye-PCR fragments were purified with QIAquick PCR Purification Kit (Qiagen) and used for the binding reaction at a concentration of around 50 nM. DNA binding reactions were performed in 20 µl of binding buffer containing 10 mM Tris-HCl pH 8.5, 50 mM NaCl, 10 mM EDTA, 0.5% Tween-20, 10 mM DTT, and 1 mg of poly(dI-dC)-labelled IRdye-PCR fragments at room temperature for 30 min. For the specific and non-specific competition assays, D(+)-glucose 6-phosphate (30 mM) (Sigma) was added to the binding buffer in the EMSA as CcpA cofactor [35]. Purified rApuR and rCcpA proteins (from 0.5 to 4.0 µM) were incubated, in separate experiments, with the fragment Pr2 and a non-specific competitor fragment that was obtained by PCR amplification of the gene SSU0879 (from 25 to 150 nM). Two 95 bp oligonucleotides complementary to Pr2 fragment lacking the two predicted binding sites (ΔOM1 and ΔOM2/*cre*) were synthesized (Eurogentec, The Netherlands), PCR-amplified with labelled Pr2F/R primers and incubated with the proteins. Free and bound DNAs were separated on 5% Tris-Borate-EDTA (TBE) native gels for 30 min at 10 cm/v. The gels were visualized using a LI-COR Odyssey Imager and scanned at 800 nm wavelengths.

### Titration of hemolytic activity

The hemolytic activity was assayed as previously described [36]. Two different independent assays were carried out in triplicate. Briefly, *S. suis* bacteria were grown in CM+Glc and CM+Pul at three different growth stages: lag (OD_600_ 0.1–0.2), exponential (OD_600_ 0.2–0.5) and stationary (OD_600_ 0.5–0.7). The supernatant was collected from 1 ml for each culture by centrifugation at 12000 *g* for 1 min. Serial twofold dilutions (150 µl) of test samples were prepared in polystyrene deep-well titer plates (Beckman) with 10 mM Tris-buffered saline (PBS, pH 7.4). Subsequently, 150 µl of a 2% washed horse erythrocyte suspension in 10 mM Tris-buffered saline containing 0.5% BSA was added to each well. After the wells were sealed, the plates were incubated on a Coulter mixer for 2 h at 37°C. Unlysed erythrocytes were sedimented by centrifugation (1500 *g* for 10 min), 150 µl portions of the supernatant were transferred to a polystyrene flat-bottom microtiter plate and measured at 540 nm with a microELISA (enzyme-linked immunosorbent assay) reader (SpectraMax M5, Molecular Devices LLC). A 100% lysis reference sample was obtained by lysing cells with 1% Triton-X and the background lysis was subtracted before calculation of hemolytic activity.

### Adherence and invasion assays using NPTr cell line

Culturing of Newborn pig tracheal cells (NPTr) [37] and adhesion assays were performed as previously described [19]. For the invasion assays, *S. suis* was added to the cell culture at an multiplicity of infection (m.o.i.) of ∼50 and incubated for 2 h at 37°C with 5% CO_2_ to allow cellular invasion by the bacteria. The monolayers were then washed three times with PBS; 1 ml of cell culture medium containing 100 µl/ml gentamycin and 5 µg/ml penicillin G was added to each well, and the plates were incubated for 2 h at 37°C, 5% CO_2_ to kill extracellular and surface-adherent bacteria. The monolayers were washed three times with PBS, and cells were disrupted by the addition of 800 µl of ice-cold Milli-Q water and repeated up-and-down pipetting to release intracellular bacteria. To enumerate the viable bacteria, serial dilutions of each cell lysate were plated in triplicate on Columbia sheep blood agar plates and incubated at 37°C for 24 h. The rate of invasion was expressed as percentage of initial inoculum that was recovered per well. Two independent assays were performed in triplicate.

### Experimental infection of pigs

The animal experiment described in this paper was approved by the ethical committee of the Central Veterinary Institute of Wageningen UR, Lelystad, The Netherlands under proposal number 2010113c in accordance with Dutch legislation (The Experiments on Animals Act, 1997) and the general principles governing the use of animals in experiments of the European Communities (Directive 86/609/EEC). To reduce unnecessary suffering of animals, humane end points were used: piglets were euthanized as soon as *S. suis*-specific symptoms occurred. Caesarean-derived, colostrum-deprived (CDCD) piglets were infected i.v. with 1×10^6^ CFU of S735-pCOM1-orf2 [23]. The strain used to experimentally infect pigs was different to that used in our *in vitro* qPCR assays but was selected because it was known to be effectively recovered from the blood and organs in experimental pig infections. Genes encoding *apuA* and *sly* are both present in the genome of this strain (not shown). Animals were euthanized when specific *S. suis* symptoms (meningitis, arthritis, sepsis) were observed, or at the end of the experiment (6 days post infection). After animals were euthanized; organs and specific sites of *S. suis* infection were examined macroscopically and bacteriologically. Tissue samples were collected during post-mortem observation and snap-frozen in liquid nitrogen.

### Quantitative PCR using bacterial-enriched RNA isolated from *in vivo* tissues and blood

Blood collected in PAXgene tubes was treated as recommended by the manufacturer (PreAnalytix/Qiagen). Samples taken from heart, brain and joints consisted of a mixed pellet of porcine cells combined with bacteria. All pellets from *in vivo* samples were thawed on ice, resuspended in 600 µl of Trizol (Invitrogen, Carlsbad, CA, USA) and subjected to 40 seconds of 6.5 m s^−2^ in the Fastprep-24 (MP Biomedicals, Solon, OH, USA) to disrupt bacteria. The mixture was extracted with 120 µl of chloroform, mixed for 15 seconds, incubated for 3 min at RT and centrifuged for 15 min at 20.000 *x g*. Supernatant was removed and extracted with 1 volume of chloroform. RNA in the supernatant was precipitated with 1 volume of isopropanol. After incubation for 30 min at RT or 16 h at −20°C, RNA was collected by centrifugation, and washed with 70% ethanol. The pellet was resuspended in water. Subsequently, RNA was purified as described above. To remove eukaryotic RNA all samples were treated using the MicrobEnrich kit (Ambion, Austin, Tx, USA). Bacterial-enriched RNA was amplified using the Ovation PicoSl WTA system v2 (Nugen, San Carlos, CA, USA). cDNA was purified using MinElute spin columns (Qiagen, Hilden, Germany) and diluted 25 times for qPCR analysis. Primers were designed using PrimerExpress software (Applied Biosystems, Foster City, CA, USA) (Table S1). Each reaction contained 12.5 pmol forward primer, 12.5 pmol reverse primer and POWR SYBR Green PCR Master Mix (Applied Biosystems). qPCR was performed using an ABI7500 (Applied Biosystems). As the bacterial RNA was amplified we used a more stringent GeNorm method [38] for normalization of the real-time qPCR data. GeNorm utilizes multiple internal control genes for normalization, in this case *gyrA*, *proS*, and *mutS* that were most stably expressed among 7 tested genes (data not shown). In each run a standard curve was incorporated consisting of seven 10-fold dilutions of a vector containing the cloned target of the PCR. In this way, both target genes and the reference genes could be related to a standard line. For each reaction, negative water controls were included. Analysis was performed using the ABI7500 Software (Applied Biosystems).

### Statistical analysis

The results obtained in the *in vitro* studies were analyzed using GraphPad Prism version 5.0 software (San Diego California, USA). All qPCR experiments and hemolytic activity assay were reproduced at least two times in triplicates and, where indicated, representative experiments are shown. Two-way ANOVA tests were carried out using Bonferroni's post hoc test. The adhesion and invasion assays were performed at least two times using triplicate samples. All numerical data presented here are expressed as means ± standard error of the mean (SEM). Statistical significance was determined using a two-tailed unpaired Student's t test. Differences were considered significant at *p*<0.05. Statistical significance was indicated as follows: * *p*<0.05; ** *p*<0.01; *** *p*<0.001.

## Results

### The switch from glucose to α-glucan starch fermentation has pleiotropic effects on gene expression

To investigate the global effects of a shift in carbohydrate metabolism on *S. suis* gene expression we compared genome-wide transcriptomic data from exponential (e) and stationary phase (s) cultures of *S. suis* in complex media (CM) supplemented with 1% of starch/pullulan (α-1,4; α-1,6 glucan) (Pul) or glucose (Glc) as carbon sources (Figure S1 and Table S2).

**Figure 1:**
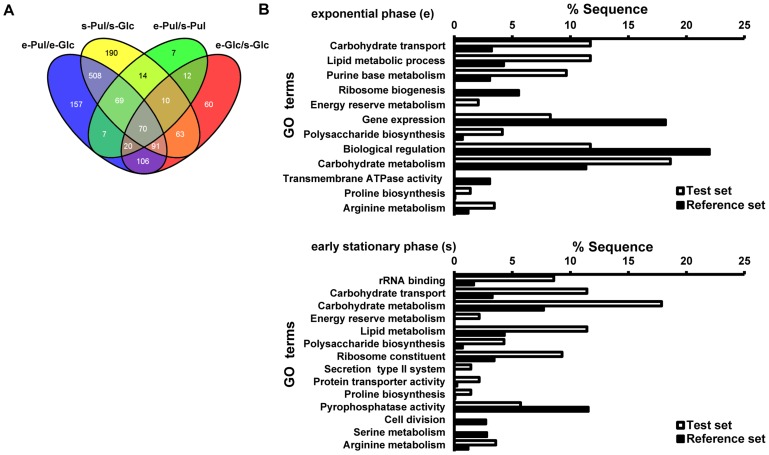
Comparison of genome-wide transcriptomic data for exponential (e) and early stationary phase (s) cultures of *S. suis* grown in pullulan (Pul) or glucose (Glc). **A**. Venn diagram of *S. suis* genes differentially regulated during growth in pullulan (Pul) vs glucose (Glc) in the exponential (e) or stationary (s) phase. In each sector the numbers of shared or unique differentially expressed genes is indicated. **B**. GO term distribution of *S. suis* genes differentially regulated in pullulan vs glucose in early exponential and early stationary phase. GO Enrichment analysis was performed using BLAST2GO (P = 0.05, two-tailed Fisher's Exact test).

The numbers of genes differentially expressed during growth in pullulan versus glucose were 1028 (52% of annotated genes) during exponential growth and 1015 (51% of annotated genes) during early stationary growth (Figure 1A). In total 738 (37% of annotated genes) genes were differentially regulated in pullulan compared to glucose, irrespective of the growth phase. In starch/pullulan, 209 genes were differentially regulated between the exponential and early stationary phases of growth; in glucose, 432 genes were differentially regulated for the same comparison (Figure 1A).

To link gene expression data to changes in bacterial metabolic and physiological pathways, we obtained gene ontology (GO) functional gene annotations of all differentially expressed *S. suis* P1/7 genes using BLAST2GO (see Methods) (Figure 1B). Similar analyses were also performed for a subset of differentials, namely all upregulated genes within the GO category “carbohydrate metabolism” (Figure S2). The highest number of genes differentially expressed in both growth phases participated in “carbohydrate metabolism” (18%) and “carbohydrate transport” (11%). Genes in the GO category “energy reserve metabolism” (2% for both growth phases) and lipid metabolism that generates the precursors of lipoteichoic acids and membrane phospholipids (11% for both growth phases) were also enriched when bacteria were grown in starch/pullulan vs glucose (Figure 1B). Other enriched GO categories included amino acid metabolic pathways for arginine and proline.

**Figure 2:**
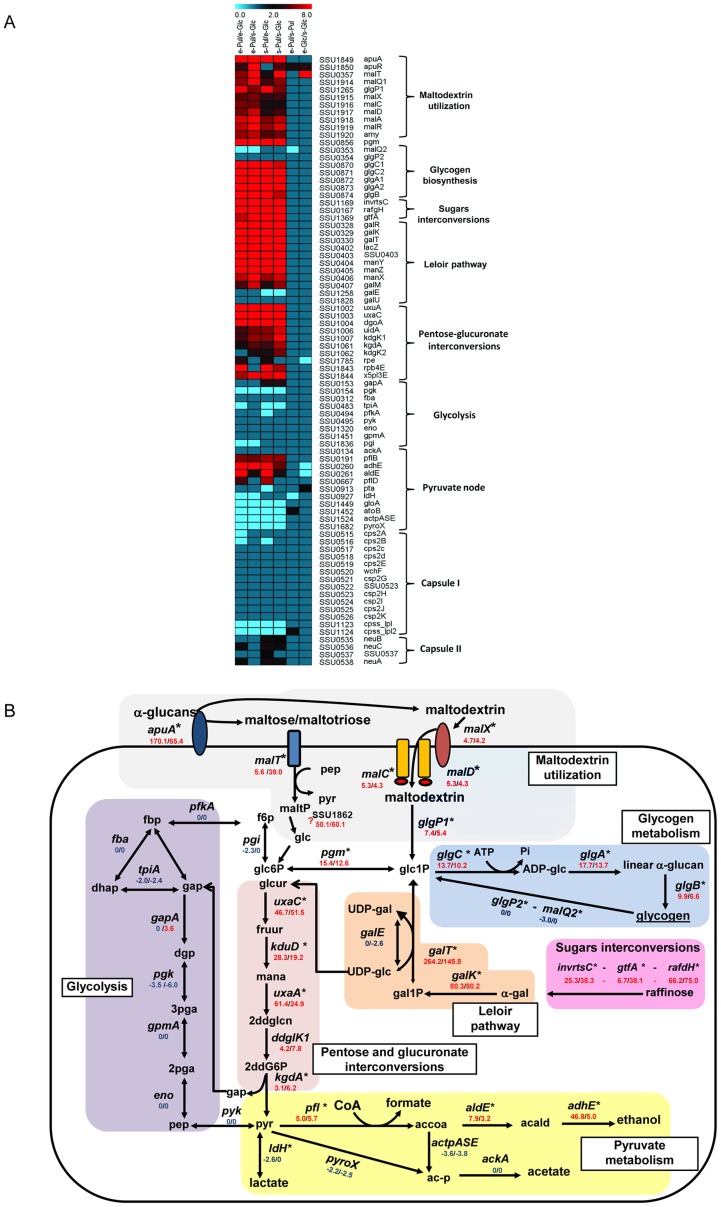
Transcriptome analysis of *S. suis* metabolism in presence of pullulan. **A**. Heatmap showing the effect of starch/pullulan on the transcription of genes involved in carbohydrate metabolism and capsule production. Expression (ratios) of genes participating in different pathways (indicated at the right of the heatmap) are shown for 6 different comparisons (indicated at the top of the heatmap). At the top of the Figure 2A, a color scale depicts the ratio of expression during growth in pullulan vs. glucose. Red indicates induction (upregulation) of the respective genes and blue indicates repression (downregulation) of the respective genes for each comparison. For each gene, the *S. suis* P1/7 locus tag and the gene name is depicted on the right. **B**. Schematic representation of *S. suis* metabolic pathways differentially regulated in pullulan vs glucose. α-glucans (i.e. starch/pullulan) are degraded by extracellular amylopullulanase (*apuA*) and the end degradation products, maltose/maltotriose and maltodextrins, are transported by PTS for maltose/maltotriose (*malT*) and maltodextrin ABC transport inside the bacteria (*malX, malC* and *malD*). Maltodextrins and maltose are most lilkely converted to glucose-1-phosphate (Glc1P) or α-glucose by 4-α-glucanotransferase and maltodextrin phosphorylase (*malQ*1 and *glgP*1 respectively). Glc1P can be metabolized in different pathways: phosphoglucomutase (*pgm*) isomerize glc1P to glucose-6-phosphate (glc6P) which may enter glycolysis (violet box) where it is consequently oxidated to pyruvate (pyr). Homolactic fermentation reduces pyruvate into lactate, whereas heterofermentative growth leads to other products, such as formate, acetate and ethanol (pyruvate metabolism, yellow box). The excess of glc1P that cannot enter in glycolysis may be used for synthesis of glycogen as an energy reserve (light blue box). The genome of *S. suis* is predicted to encode the enzymes sucrose phosphorylase *gtfA*, α-fructofuranosidase (interconvertase) *invrtsC*, and raffinose galactohydrolase, *rafgH* for the interconversion of raffinose- like sugars. These enzymes participate in the starch and galactose Leloir pathway. Part of Leloir pathway (e.g. galactose-1-phosphate uridylyltransferase, *galT*, and galactokinase, *galK*) was induced more strongly in starch/pullulan. *GalT* interconverts galactose-1-phosphate (gal1-P) and UDP-Glucose (UDP-glc) to UDP-galactose (UDP-gal) and glc1P. Alternatively, UDP-glc may be converted into glucuronic acid (glcur) by UDP-D-glucuronate (UDP-glcur) to enter in an alternative (to glycolysis) pathway for pyruvate (pyr) production. Pathway predictions were reconstructed based on genome information, literature and database surveys (KEGG, MetaCyc). The following gene annotation was downloaded from NCBI: *galM*, aldose 1-epimerase; *galK*, galactokinase; *galE*, UDP-glucose 4-epimerase; *galT*, galactose 1-phosphate uridylyltransferase; *pgm*, Phosphoglucomutase/phosphomannomutase; *pfkA*, 6-phosphofructokinase; *fba*, fructose bisphosphate aldolase; *tpiA*, triosephosphate isomerase; *gapA*, glyceraldehyde-3-phosphate dehydrogenase; *pgk*, phosphoglycerate kinase; *gpmA*, phosphoglyceromutase; *eno*, phosphopyruvate hydratase; *pyk*, pyruvate kinase; *ldh*, L-lactate dehydrogenase; *pyroX*, pyruvate oxidase; *ackA*, acetate kinase; *pfl*, pyruvate formate-lyase; *adlE* acetaldehyde-CoA dehydrogenase; *adhE* alcohol dehydrogenase; *glgB*, *glgA* glycogen synthase; *glgC* glucose-1-phosphate adenylyltransferase; *glgP* glycogen phosphorylase.

These analyses showed that culturing *S. suis* in presence of these different carbon sources leads to changes of 35–50% of the *S. suis* transcriptome, affecting not only carbohydrate metabolism but also basal metabolic and stress survival pathways. To summarize these broad changes in global transcriptomes, we generated a visual representation of the main carbohydrate pathway genes for six growth conditions comparisons according to their expression ratios (see Methods) (Figure 2A).

Comparative transcriptome analysis of *S. suis* grown in starch/pullulan vs glucose revealed that growth in pullulan induced expression of the maltodextrins utilization gene cluster that includes *apuA* and the corresponding phosphotransferase systems (PTS) and ATP-binding cassette (ABC) transporters. The expression of the glycolysis pathway genes did not change significantly during growth in pullulan compared to glucose, suggesting that activity of this pathway was not altered during growth in both carbon sources. Rather, it appeared that excess intracellular glucose was converted in glycogen as energy reserves (glycogen biosynthesis pathways) or had entered into hexose-pentose carbohydrate pathways as an alternative to glycolysis (sugar interconversion and Leloir pathways) (Figure 2A). The biochemical links between the differentially expressed metabolic pathways are shown in Figure 2B; for each pathway, an extensive overview of the main genes involved and their functions can be found in the SI Text S2.

### Links between carbohydrate metabolism and virulence gene regulation

We analyzed the transcriptome data to see whether the differential fermentation of starch/pullulan and glucose affected expression of known or predicted *S. suis* virulence genes. Seven genes possibly involved in the invasion of mucosal tissues or avoidance of host defenses by streptococcal pathogens were highly upregulated (expression ratio>10) in starch/pullulan compared to glucose (Table 1, Figure S3, Text S2). As CcpA in Gram-positive bacteria controls carbohydrate metabolism, one of the most profoundly altered categories in our microarray experiment, we investigated the potential role of CcpA in regulating virulence gene expression. We first used the MEME [39] and MAST [33] algorithms to mine the genome sequence of *S. suis* P1/7 for catabolite-responsive element (*cre*) sites, short DNA regions that can be bound by CcpA, using the consensus *B. subtilis cre* sequence [40]–[42].

**Table 1:** Confirmed and proposed *S. suis* virulence factors differentially expressed in pullulan (Pul) compared to glucose (Glc).

Annotation *S. suis* P 1/7	Protein	Function	Virulence	Pul/Glc^1^	Biblio
Galactosyl/rhamnosyl transferase - SSU0520	CpsE/F	CPS biosynthesis	Attenuated-pig		[71]
Tyrosine-protein kinase Wze - SSU0517	Cps2C	CPS biosynthesis	Attenuated-pig		[71]
N-acetylneuraminic acid synthase - SSU0535	NeuB	Sialic acid synthesis	Attenuated-pig	U	[72]
Peptidoglycan GlcNAc deacetylase - SSU1448	PgdA	Peptidoglycan	Attenuated-pig	D	[73]
D-alanine-poly ligase - SSU0554	DltA	LTA D-alanylation	Attenuated-pig		[74]
Fibronectin-fibrinogen binding - SSU1311	FbpS	Adhesion ECM	Attenuated-pig		[75]
Enolase - SSU1320	Eno	Adhesion ECM	no Mutant		[76]
Glyceraldehyde-3-P-dehydrog - SSU0153	GAPDH	Adhesion ECM	no Mutant	U	[77]
Di-peptidyl peptidase IV - SSU0187	DppIV	Adhesion ECM	Attenuated-mouse	U	[78]
Amynoacyl histidine peptidase - SSU1215	PepD	Subtilisin- protease	No Mutant	U>10	[79]
6-phosphogluconate-dehydrogen - SSU1541	6-PGD	Adhesion epithelium	No Mutant		[80]
Amylopullulanase - SSU1849	ApuA	Adhesion epithelium	Not tested	U>10	[19]
Glutamine synthetase - SSU0157	GlnA	Adhesion epithelium	Attenuated-mouse	D	[81]
Streptococcal adhesin P - SSU0253	SadP	Adhesion epithelium	no Mutant	U	[82]
Arginine deaminase - SSU0580	ArcB	Resistance to acidity	Not tested	U>10	[83]
Anchored DNA nuclease - SSU1760	SsnA	DNA degradation	Not tested	U	[84]
Cell envelope proteinase - SSU0757	SspA	Subtilisin- protease	Attenuated-mouse	U	[85]
Metallo-serine protease - SSU1773	IgAP	IgA1 protease	Attenuated-pig	U>10	[86]
Suilysin - SSU1231	Sly	Haemolysin	Unaffected-pig	U>10	[36]
Hyaluronate lyase - SSU1050	Hyl	hyaluronidase	Not tested	U>10	[87]
putative oligohyaluronate lyase - SSU1048	HepI/III	hyaluronidase	Not tested	U>10	[88]
Sortase A - SSU0925	SrtA	Protein sorting	Attenuated-pig		[89]
Serum opacity-like factor - SSU1474	OFS	Serum opacification	Attenuated-pig	D	[90]
S-ribosyl homocysteinase - SSU0376	LuxS	Quorum sensing	Attenuated zebrafish	D	[91]
Muramidase released protein - SSU0706	MRP	Unknown	Unaffected-pig	U	[22]
Extracellular protein factor - SSU0171	Ef	Unknown	Unaffected-pig	U	[92]

Pul/Glc1 upregulated (U) or downregulated (D) expression when *S. suis* was grown in pullulan (Pul) compared to glucose (Glc).

38 potential *cre* sites located upstream of the start codon of predicted proteins (P) or in proximity of the gene transcription start site (G) were predicted to control expression of 172 genes through interaction with CcpA (Table S3). Of the 172 genes in the predicted CcpA regulon, 145 (84%) were differentially regulated during growth in starch/pullulan compared to glucose (Table S3). In Table S3 we show 38 *cre* sites and their downstream genes or operons and if these were differentially expressed in pullulan compared to glucose (our study) or in an *S. suis* serotype 2 Δ*ccpA* mutant compared to wild-type [43].

As expected, *cre* sites were commonly associated with the predicted promoters of carbohydrate PTS and ABC transporters (17%) and enzymes for carbohydrate metabolism (25%). Additionally, *cre* sites were identified in the promoters of regulators (10%) and 9 (8%) out of the 19 virulence genes (47%) that were differentially expressed in pullulan compared to glucose, including *sly* and *apuA* (Table S3). As *apuA* is essential for growth on pullulan and was shown to play a role in colonization of mucosal epithelia *in vitro* [19] and suilysin is a major virulence factor, we sought to understand the regulation of the encoding genes in more detail and explore the role of CcpA in transcriptional regulation during the switch from glucose to starch fermentation.

### Expression of *apuA* and *sly* is repressed by CCR

**Figure 3:**
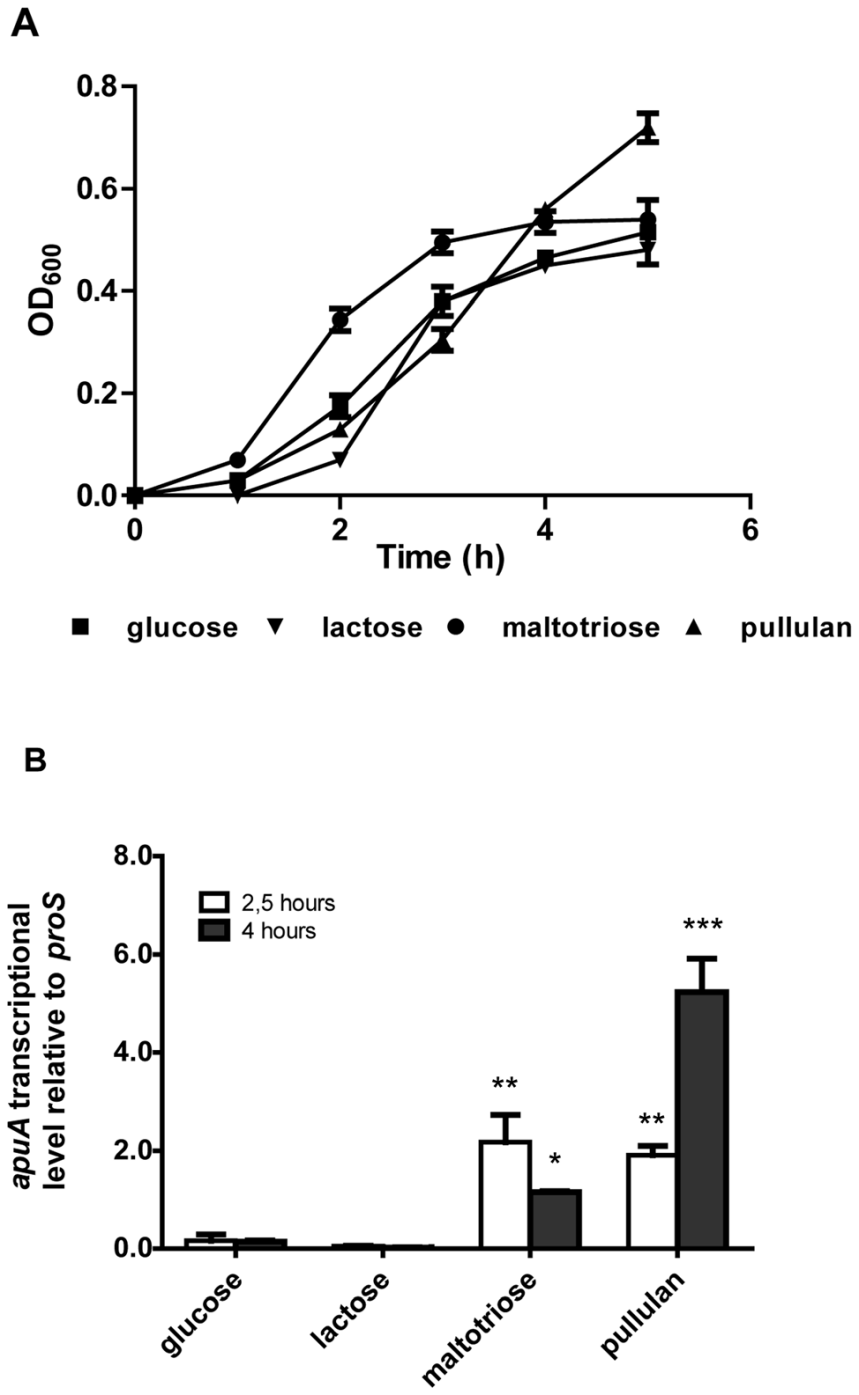
Transcriptional regulation of *apuA* and *sly* grown in the presence of different carbon sources. **A**. *S. suis* S10 growth curve at 37°C in CM containing 1% (w/v) of different sugars as indicated. The graph shows the means and standard deviations from two independent experiments. **B**. and **C**. Relative expression of *apuA* and *sly* in *S. suis* grown in CM containing 1% (w/v) different sugars was determined by qPCR. The transcript levels of *apuA* were measured after 2.5 hours and 4 hours of growth relative to the reference gene *proS*, which is constitutively expressed at similar levels during growth in different sugars (data not shown). The height of the bars represent mean values for the relative expression data ± SEM from 2 independent experiments (n = 3). Statistical significance was calculated using a two-way ANOVA test followed by Bonferroni's post hoc test (* *p*<0.05; ** *p*<0.01; *** *p*<0.001.).

We assessed the relative expression of *apuA* and *sly* by qPCR when *S. suis* was grown in complex media supplemented with glucose, lactose, starch/pullulan or maltotriose, all of which supported efficient growth (Figure 3 panels A). Compared to growth in glucose or lactose, *apuA* and *sly* transcription was induced by growth in pullulan (up to 5.2 and 1.3 fold after 4 hours respectively; P<0.001) and maltotriose (up to 2.2 and 0.23 after two 2.5 hours respectively; P<0.01) (Figure 3 panels B and C).

**Figure 4:**
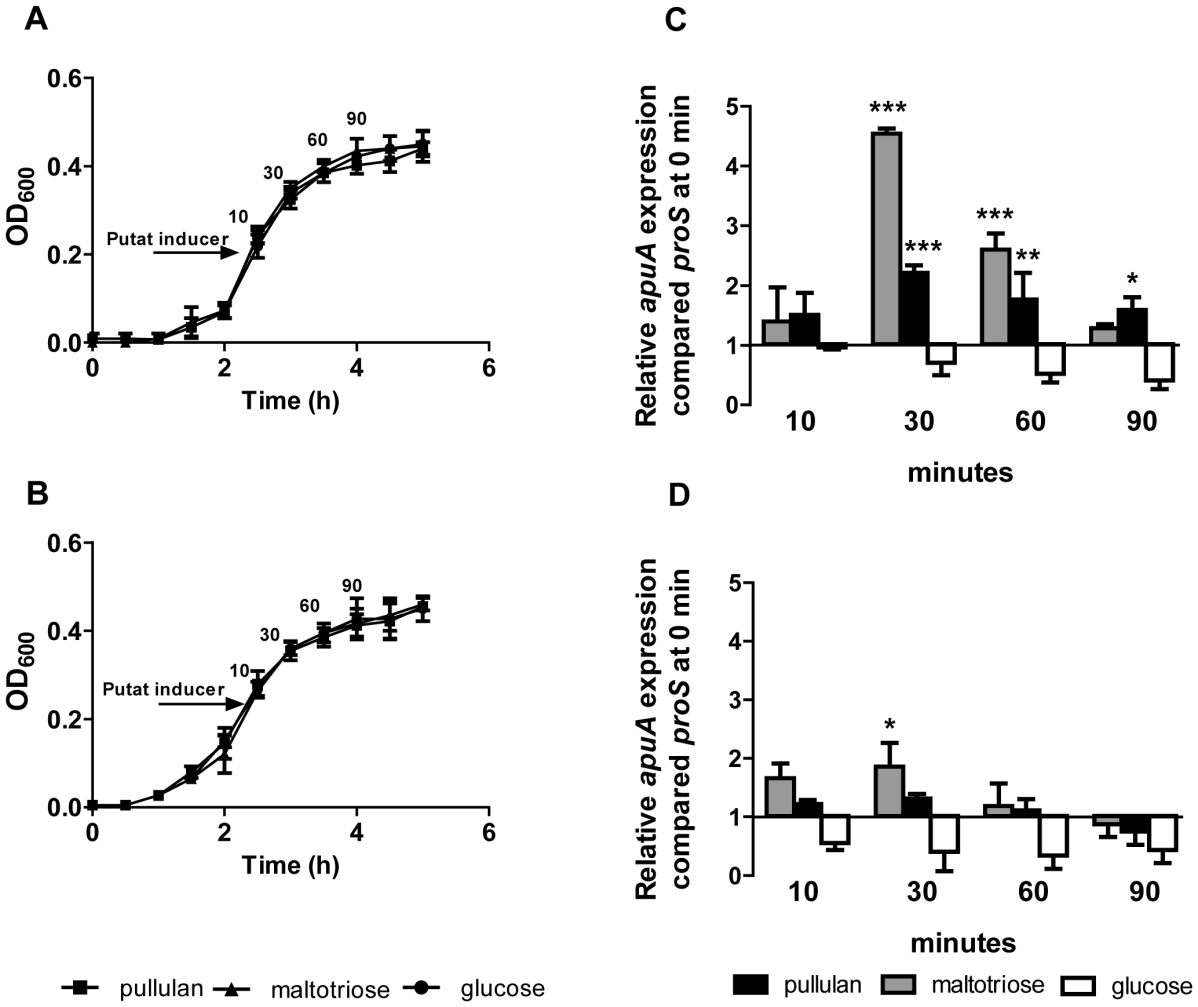
Induction of *apuA* expression by putative inducers in the presence of glucose or lactose. **A**. Growth curves of *S. suis* S10 in CM containing 1% w/v lactose or **B**. 1% w/v glucose before and after addition of 0.25% w/v putative inducers (arrow) i.e. maltotriose, pullulan or glucose. The graphs show the means and standard deviations from two independent experiments. **C**. Relative expression of *apu*A genes following addition of putative inducers to *S. suis* growing in CM plus lactose. **D**. Relative expression of *apu*A genes following addition of putative inducers. The relative expression of *apuA*, was measured by qPCR 10, 30, 60 and 90 minutes after addition of the putative inducers, The height of the bars shows the mean (n = 3) fold change in expression ± SEM from two independent experiments. Statistical significance was calculated using a two-way ANOVA test followed by Bonferroni's post hoc test (* *p*<0.05; ** *p*<0.01; *** *p*<0.001.).

As *apuA* and *sly* contain a conserved *cre* in the promoter region (Table S3), we predicted that their transcription might be repressed during growth on glucose but not lactose. However, the relative level of *apuA* transcription in lactose was comparable to that of transcription in glucose suggesting possible regulation by a second transcriptional regulator. To test this hypothesis we added starch/pullulan or maltotriose to *S. suis* growing exponentially in either lactose (Figure 4A) or glucose (Figure 4B) and quantified *apuA* transcription at different time points (Figure 4 panels C and D). In medium containing lactose, both maltotriose and pullulan strongly induced expression of *apuA* after 30 min (4.5 fold and 2.2 fold respectively; P<0.001; Figure 4C). In medium containing glucose the addition of maltotriose or starch/pullulan had a small effect on the expression of *apuA*; only a slight increase (1.6 fold; P<0.05) of *apuA* expression was observed 30 min after addition of maltotriose (Figure 4D). These results show that when *S. suis* is growing in glucose for 30 min, *apuA* transcription is hardly induced by pullulan or maltotriose but when grown in lactose for 30 min, *apuA* expression is strongly induced by pullulan and maltotriose.

### CcpA and ApuR bind to conserved operator motifs in the *apuA* promoter

**Figure 5:**
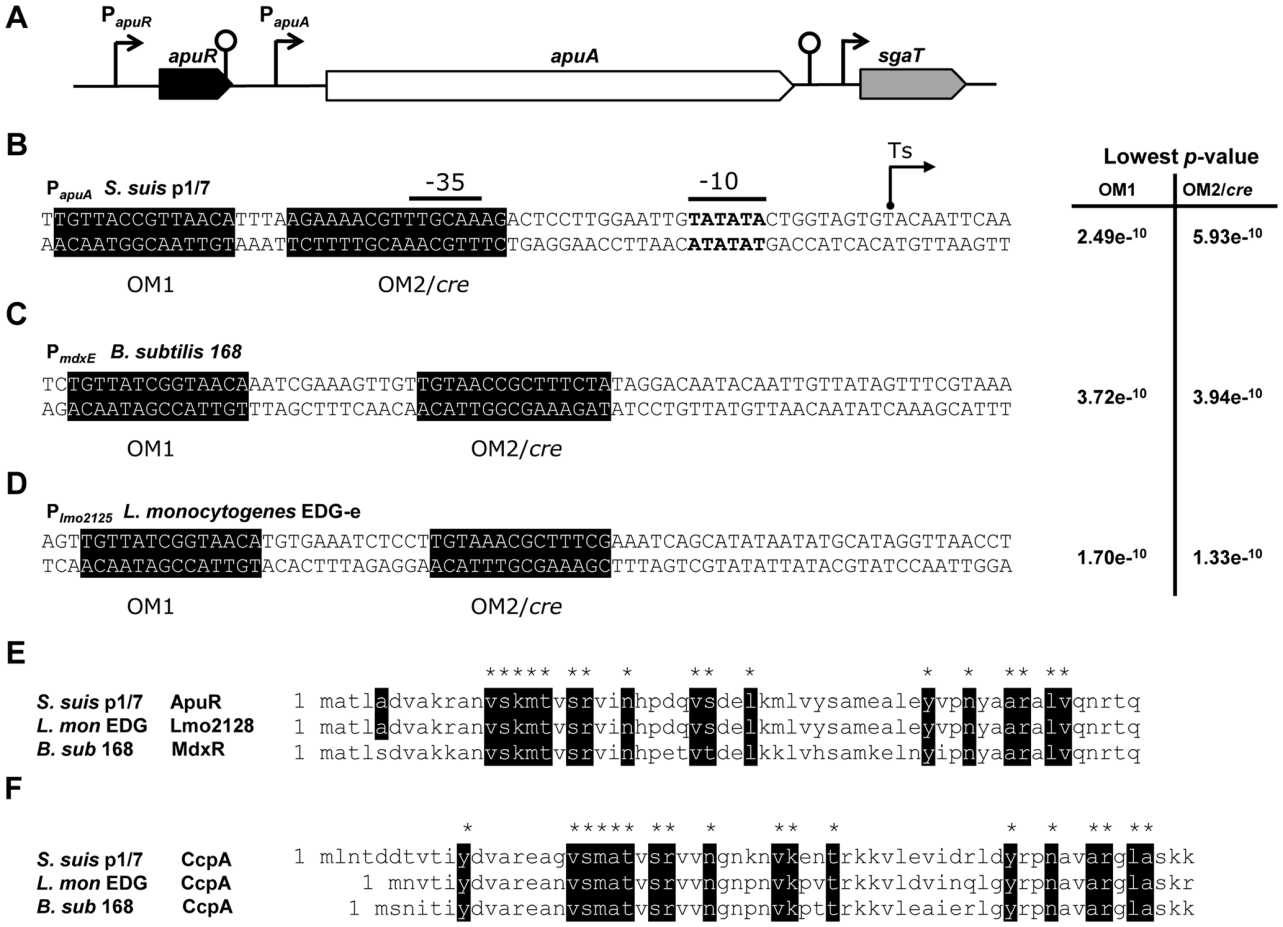
Identification of conserved operator binding motifs for ApuR (OM1) and CcpA (OM2) in *S. suis* P1/7, *B. subtilis* 168 and *L. monocytogenes* EDG-e. **A**. The 6 kb amylopullulanase gene *apuA* is located downstream of *apuR* which encodes a putative transcriptional regulator of the LacI/GalR family. Located downstream of *apuA* are a cluster of genes predicted to be involved in uptake and fermentation of ascorbate (*sgaT*, *sgaB*). For each gene, the direction of transcription is indicated by an arrow, the size of which is proportional to the length of the corresponding open reading frame. Putative promoters are represented by arrows and transcription terminators by loops. The operator motifs OM1 and OM2/cre (shaded sequences in B, C and D) were determined using the MEME software suite and their relative probability p-values are indicated. **B**. The apuA promoter based on the experimentally determined transcription start site (Ts arrow) **C**. The *B. subtilis mdxE* (BSU34610) promoter **D**. The *L. monocytogenes lmo2125* promoter **E**. and **F**. Sequence alignment of the DNA binding domains of the ApuR and CcpA proteins of *S. suis, L. monocytogenes* and *B. subtilis*. Conserved amino acid sequences are indicated in black.

We hypothesized that the induction of *apuA* expression by maltotriose was due to ApuR, a putative transcriptional regulator upstream of *apuA* that possesses its own promoter and a predicted rho-independent downstream terminator (Figure 5A). Homology searches indicated that ApuR was a LacI/GalR type regulator containing an N-terminus DNA-binding and a C-terminus ligand-binding domain that can be bound by a specific sugar. Comparison of ApuR with protein sequences in the UniProt database revealed similarities to several transcriptional regulators of operons (Figure S4) which comprising maltodextrin utilization gene clusters. In the top ranking were listed for highest similarities: BL23 YvdE (*Lactobacillus casei*; 53% identity) [44], MdxR (syn. YyvdE; *B. subtilis* 168; 49% identity) [45], [46], the activator EGD-e Lmo2128 (*Listeria monocytogenes*; 47% identity) [47] (Figure 5 E–F) and the activator MdxR (*Enterococcus faecium* E1162; 49% identity) [48].

As *apuA* is considered a relevant virulence factor and its regulation by different carbohydrates could be of biologic relevance, we decided to investigate the binding of CcpA and ApuR to operator sites in the *apuA* promoter. To elucidate the *apuA* promoter regulatory modules we determined the transcription start site, conducted homology searches for binding motifs of known regulators *in silico*, and performed *in vitro* promoter binding assays with purified transcription factors (TFs). The transcriptional start site of the *apuA* transcript was determined experimentally by 5′-RACE to be 31 nt upstream of the start codon. The consensus −35 element (5′-TTGCAA-3′) for RpoD (sigma 70) and the −10 element (5′-TATATA-3′) required for interaction with the RNA polymerase and transcription initiation were found near the expected positions upstream of the transcription start site (Figure 5B). Visual inspection of the *apuA* promoter region revealed the presence of two potential operator motifs (OMs) that were also listed in the RegPrecise database [34]. One *mdxR*-like operator motif, designated here as OM1, was found 13 bp upstream of the −35 element; a second operator motif predicted *cre* site designated OM2/*cre*, located at position −30 nt, overlapping the conserved −35 element; this overlap could interfere with binding of the RNA polymerase (Figure 5 C–F).

To demonstrate binding of ApuR and CcpA to the predicted operator motifs, we expressed and purified these proteins in *E. coli* with a C-terminal His-tag (Figure S5 and B) and tested DNA binding in electrophoretic mobility shift assays (EMSA) using three overlapping fragments of the P*_apuA_* promoter region (Pr 1–3; approx. 120 bp in length). A single rApuR-DNA complex was observed with promoter fragments Pr2 and Pr3 suggesting the binding motif for ApuR lies within the 64 bp overlapping region (Figure S6 panels A and B). The binding of ApuR to Pr2 was shown to be concentration dependent in the range from 0.5 to 4.0 µM of rApuR (Figure S6C). The specificity of rApuR binding to Pr2 was shown in a competition EMSA using unlabeled competitor DNA (i.e. fragment Pr2) or an unlabeled non-specific competitor lacking the two predicted OM1 and OM2/*cre* operator binding motifs. The non-specific competitor DNA fragment had no effect on Pr2 complex formation whereas the specific competitor (Pr2) substantially reduced rApuR binding (Figure S6 panels D and E). To investigate whether ApuR binds to OM1 or OM2/*cre*, promoter fragments lacking these motifs were synthesized and tested in the EMSA (i.e. ΔOM1 and ΔOM2/*cre*, Table S3 and S4). In three independent experiments we observed a lower amount of the rApuR-DNA complex with fragments lacking ΔOM1 suggesting that ApuR binds most strongly to this motif. In some experiments, complex formation was slightly reduced when ΔOM2/*cre* was deleted, possibly due to low affinity binding of ApuR to ΔOM2/*cre* at higher protein concentrations (Figure S6F).

Similarly, binding of CcpA to the P*_apuA_* OM2/*cre* that overlaps with the predicted −35 promoter element was demonstrated by EMSA using a fluorescent IRdye-Pr2 fragment containing the putative *cre* site (Figure S6 panels G–J). An increasing amount of an rCcpA-DNA complex was observed with an increasing concentration of purified rCcpA (1.5 to 5 µM) (Figure S6G). The complex could be outcompeted by addition of unlabeled Pr2 but not with non-specific competitor DNA indicating that CcpA binds specifically to P*_apuA_* (Figure S6 panels H and I). Recombinant CcpA also appears to bind to the ΔOM2/*cre* fragment although less DNA/transcription factor complex is observed suggesting that CcpA may also bind OM1 or other sequences in Pr2 with lower affinity (Figure S6J).

Taken together, these results show that *apuA* expression may be regulated via repression through CcpA-mediated carbon catabolite control and via transcriptional activation through a dedicated regulator encoded by the *apuR* gene.

### Relief from CCR increases adhesion and invasion of *S. suis* to porcine epithelial cells

**Figure 6:**
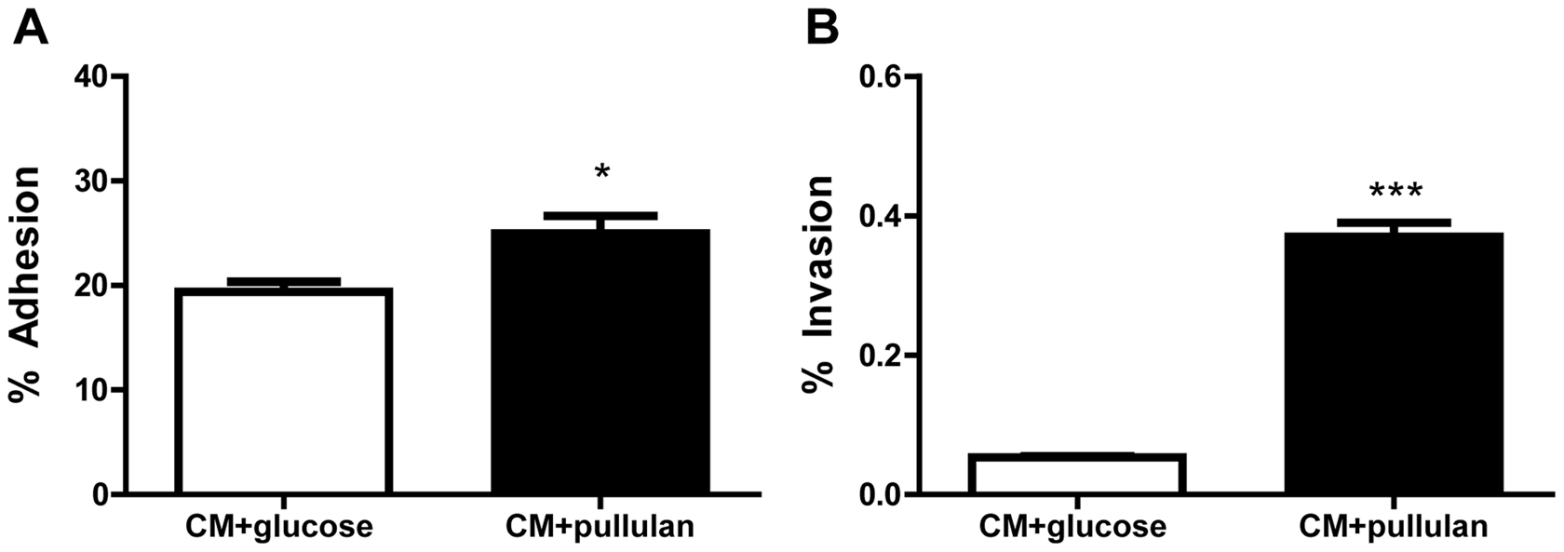
Comparison of adherence A. and invasion B. of *S. suis* after growth in CM+1% w/v pullulan (black bars) vs. CM+1% w/v glucose (white bars). NPTr confluent monolayers were co-cultivated for 2 h with *S. suis* S10 bacteria grown in CM plus pullulan or glucose. Adherence and invasion are shown as mean % values of the initial inoculum from two independent experiments in triplicate. Error bars indicate the SD.

As our microarray data showed increased transcription of *apuA* and seven other genes predicted to play a role in *S. suis* adhesion and invasion in starch/pullulan compared to glucose, we hypothesized that culturing *S. suis* in pullulan as single carbon source, thus in absence of glucose might increase its adhesion and invasion capacity. Exponentially growing *S. suis* grown in CM supplemented with 1% of glucose or pullulan were incubated with NPTr cells for 2 h at m.o.i. of ∼50 bacteria/cell. To maintain similar conditions during the period of co-culture, the cell culture medium was replaced with glucose-free DMEM supplemented with either 1% glucose or pullulan during the 2 h incubation with *S. suis*. In agreement with previous adhesion studies using the NPTr cell line [19], we found that adherence of *S. suis* bacteria grown in CM plus glucose was 19.4±1.0% of original inoculum (averaged over 3 independent replicates). The adherence of *S. suis* was significantly increased (24.9±1.7%; P<0.05; 3 replicates) after growth in 1% pullulan (Figure 6A). In accordance with previous studies using a human Hep-2 cell line [49], *S. suis* 2 S10 showed low invasion capacity (0.05% of original inoculum) in glucose. Invasiveness of *S. suis* was nearly 9-fold higher when grown in pullulan (0.40%±0.01) (P<0.01) (Figure 6B), corresponding to approximately 8.0×10^2^ cfu/ml when grown in glucose, to 7.2×10^3^ cfu/ml in pullulan. In conclusion, *S. suis* grown in starch/pullulan showed a small but significant increase in adherence to, and a strongly increased invasiveness of NPTr cells compared to *S. suis* grown in glucose.

### Carbon catabolite control of suilysin expression

**Figure 7:**
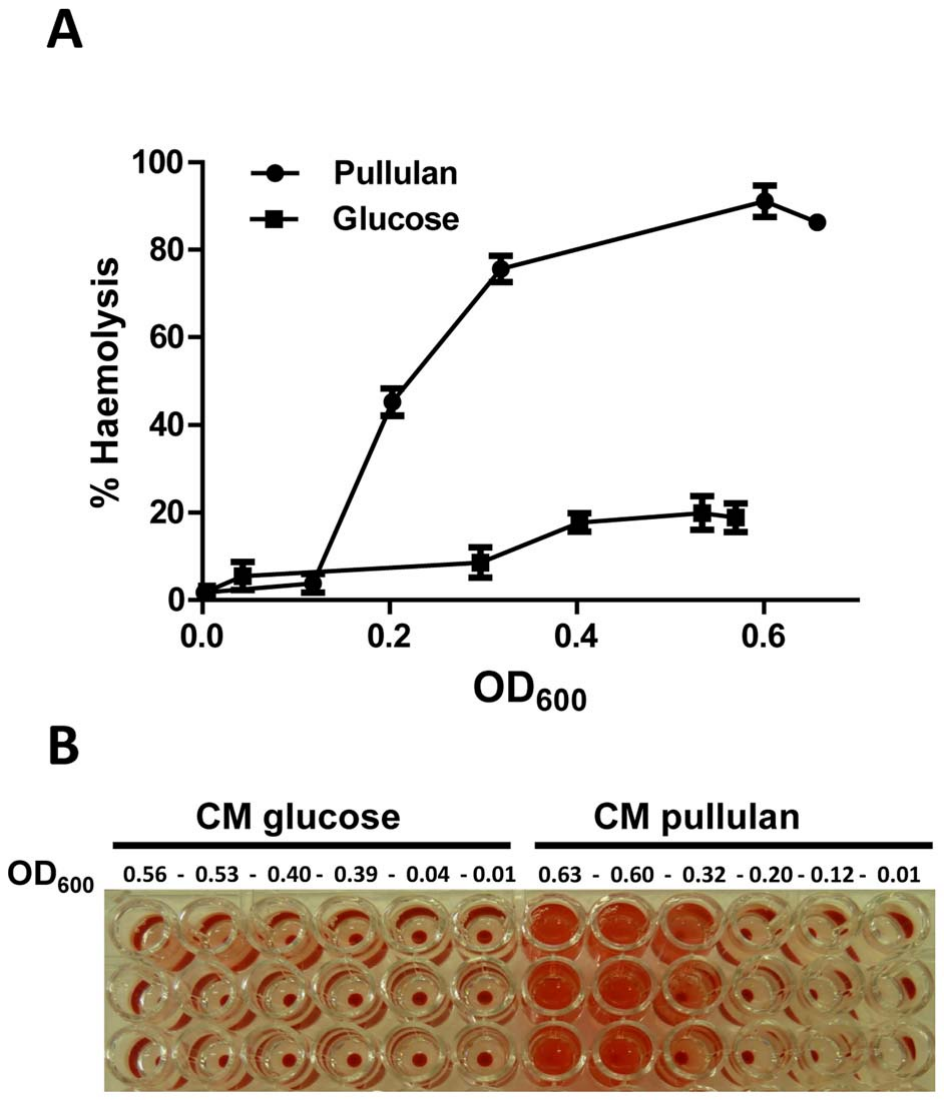
Hemolysis assay of *S. suis* growing in two different carbon sources. **A**. The hemolysis production was measured by analyzing the supernatant of *S. suis* grown in CM plus 1% w/v of glucose or pullulan in the lag, exponential and stationary phase (OD_600_ values 0 to 0.56). **B**. Deep-well titer plate showing hemolytic activity of supernatants collected from *S. suis* grown in CM supplemented with glucose or pullulan.

The expression of *sly* was strongly induced in pullulan compared to glucose, both in exponential and stationary phase (+18.1/+17.9 ratio in pullulan vs glucose). The presence of a *cre* site in the *sly* promoter region suggested that the induction of *sly* during growth in pullulan was due to relief from CcpA-mediated carbon catabolite repression rather than a specific induction by starch/pullulan. As suilysin has been proposed to compromise the integrity of the host epithelium and facilitate entry into the body we measured erythrocyte hemolytic activity (HA) of culture supernatants of *S. suis* grown in glucose or pullulan. HA was significantly higher in supernatants of S. *suis* grown in pullulan compared to glucose over a wide range (ca. 0.5) of OD600 values (P<0.001; Figure 7). In early stationary phase, HA for pullulan and glucose cultures was approx. 91% and 18%, respectively (Figure 7).

### *In vivo* expression of the virulence factors *apuA* and *sly*

**Figure 8:**
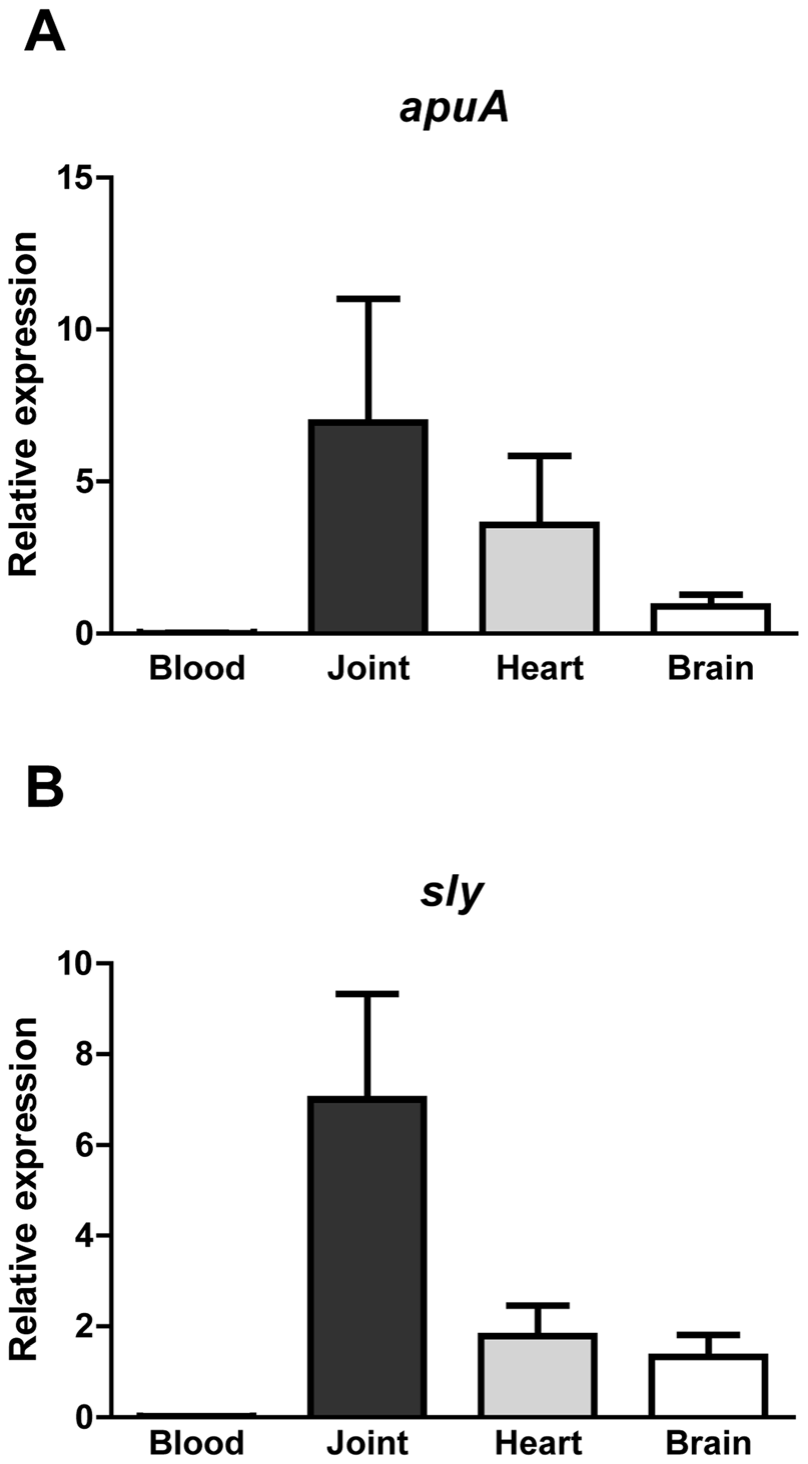
Expression of *apuA* and *sly in S. suis recovered from blood and tissues of experimentally infected piglets*. The relative expression of *apuA* and *sly* in *S. suis* blood, joints, heart and brain recovered from intravenously infected piglets calculated using the GeNorm method [38] using three housekeeping genes for data normalization. **A**. The relative expression of apuA (×10^7^) are shown for *S. suis* recovered from blood and different organs. **B**. Relative expression of sly (×10^5^) in blood and different organs.

**Figure 9:**
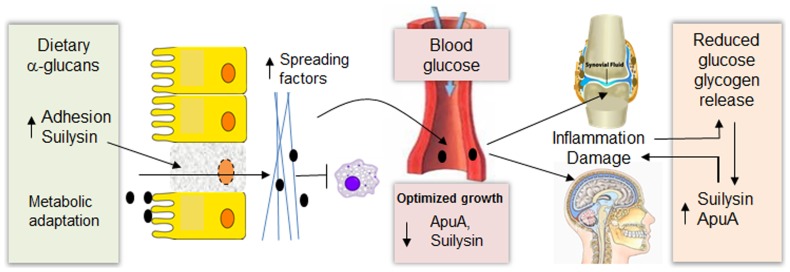
Links between carbohydrate metabolism and virulence in *Streptococcus suis*. At the mucosal surfaces a high ratio of a-glucans to glucose upregulates expression of several sugar transport systems and metabolic pathways associated with starch metabolism. Additionally, several virulence factors involved in adherence to host cells, degradation of connective tissue (spreading factors), and avoidance of phagocytic killing, including ApuA and suilysin are upregulated when glucose is diminished. Suilysin may facilitate dispersion of bacteria in mucosal tissues due to loss of barrier integrity. Once *S. suis* reaches the bloodstream metabolism is adapted for optimal growth on glucose and the expression of virulence factors is reduced by CcpA mediated-repression. In infected organs glucose levels are lower than in the blood and are further reduced by inflammation and utilization by *S. suis* leading to upregulation of ApuA, suilysin and other virulence factors. In the organs and tissues, glycogen released from damaged cells is degraded by ApuA to generate maltodextrins which sustain growth of *S. suis*.

Based on the results from this study so far, we predicted that expression of *apuA* and *sly* would be substantially higher in the mucosal colonization stage of the infection when glucose is scarce than in the bloodstream where glucose levels are sufficient to support growth and induce CCR. *S. suis* invasive disease often leads to infection of the joints, heart and brain. To test our hypothesis *S. suis* was recovered from the blood, synovial joints, heart and brains of infected piglets immediately after euthanasia and RNA was extracted from the blood and organs. As hypothesized we found transcription of *sly* and *apuA* to be significantly higher in the synovial joints, heart and brain than in the blood (Figure 8). These results show that our regulatory model (Figure 9) was informative for some aspects of porcine infection with *S. suis*.

## Discussion

Asymptomatic carriage of *S. suis* is common in adult pigs but young piglets that become colonized by *S. suis* may develop invasive disease. Host susceptibility to *S. suis* infection may occur due to inadequate host immune responses but may also be due to environmental factors such as the availability of glucose or other carbohydrates. Here we investigated whether the availability of different types of carbohydrates, notably starch and glucose, could influence the switch from an asymptomatic to a pathogenic association of *S. suis* with the host. To search for the mechanism controlling such a switch, we initially performed an *in vitro* transcriptomics study on *S. suis* grown in CM medium supplemented with pullulan or glucose; the latter mediates carbon catabolite control in streptococci via catabolite control protein A (CcpA). Pullulan, a starch, was chosen since we had previously found that growth of *S. suis* in presence of this carbohydrate induces expression of *apuA*, a gene known to be involved in carbohydrate metabolism [19].

A switch from growth in glucose to starch/pullulan resulted in a change of ca. 50% of its transcriptome, involving multiple GO function categories (Figure 1). As anticipated many genes annotated with GO term “carbohydrate metabolism” were differentially expressed, including the α-glucan-starch-degrading amylopullulanase *apuA* [19] and downstream pathway genes required for maltodextrin metabolism (Figure 2).

In other streptococcal pathogens CcpA and CCC have been shown to play a role in virulence gene expression as well as colonization [50], [51] and virulence in an animal model [51]. To predict which *S. suis* genes might be regulated by CCC we searched for conserved *cre* sites in the genome of *S. suis* P1/7 and identified 172 genes, some of which were organized in operons (Table S3). Of these 172 genes, 145 (84%) were differentially regulated during growth in pullulan compared to glucose. The difference in these two values may be due to incorrect *cre* site predictions or to the fact that expression of certain genes or operons are controlled by additional transcription factors, for example suilysin may be also co-regulated by the two-component system CovS/CovR (SSU1190-SSU1191) [52]. The 145 genes that appeared to be controlled by CCC were also compared to a published microarray data set generated by comparison of an *S. suis* serotype 2 Δ*ccpA* mutant with the corresponding wild-type strain [43]. In total, 99 genes comprising 38 operons were differentially regulated in both datasets (68% overlap; Table S3).

The CCC-regulon included genes encoding transcriptional regulators, carbohydrate metabolic enzymes and, importantly, putative or known virulence factors (8%) (Table S3). The most highly up-regulated virulence gene in pullulan compared to glucose was *apuA* encoding a cell-surface amylopullulanase required for growth on starch or glycogen that also contributes to adherence to porcine epithelial cells *in vitro* and thus may be relevant to mucosal colonization [19]. Expression of *apuA* was highly induced during growth in starch/pullulan or maltotriose, the most abundant degradation product of pullulan, and repressed during growth in glucose (Figure 3 and 4). An analysis of the promoter region of *apuA* revealed the presence of two transcription binding motifs (Figure 5) and EMSA studies showed that *apuA* expression was co-regulated by CcpA and a second regulator, ApuR (Figure S6). The similar location of the conserved OM in the promoters regulated by ApuR and the known transcriptional activator of maltodextrin utilization cluster Lmo2128 in *L. monocytogenes* [47] and MdxR in *E. faecium* [48], with high sequence similarity to ApuR, suggested that ApuR might also be a transcriptional activator. Addition of maltotriose to *S. suis* exponentially growing in medium containing lactose as a carbon source, significantly induced expression of *apuA* and, to a lesser extent, *apuR* suggesting that ApuR, like Lmo2128, might be allosterically regulated by maltotriose (Figure 4). In contrast, CcpA binding repressed *apuA* transcription, most likely through binding to an OM2/*cre* site overlapping with the −35 promoter element, since addition of maltotriose to *S. suis* bacteria precultured in glucose as sole carbon source did not lead to significant induction of *apuA*. This dual regulatory model postulates that *apuA* is maximally expressed when glucose level is low, thus allowing relief from CcpA-mediated catabolite repression, and when substrates that can be degraded by *apuA* are present.

In addition to *apuA*, eighteen other predicted or known virulence genes were differentially regulated in pullulan vs glucose, seven of which were highly upregulated in pullulan compared to glucose (Table 1, Figure S3). One of these genes was *sly* encoding suilysin, a pore-forming toxin which was secreted in 5-fold higher amounts in pullulan compared to glucose (Figure 7); its increased expression was confirmed in qPCR assays (Figure 3). Suilysin plays an important role in damaging host epithelial [12], [13], [53], endothelial [54], [55] and immune cells [56], suggesting that suilysin could play roles *in vivo* in damaging and penetration of different cell and tissue types, promoting tissue invasion and inhibition or killing of leukocytes [3], [57]. Of note, virulent *S. suis* strains that do not produce Sly may still be invasive in pigs, possibly best exemplified by Allen *et al* (2001) [58] who reported that an isogenic *S. suis sly* mutant, lacking of the hemolytic characteristics, was nearly as invasive as the wild-type parental strain in a pig infection studies after bacteria intravenous injection. The authors of the study by Allen *et al* (2001) [58] proposed that Sly is relevant for translocation across epithelia and during the infection stages prior to dispersion via the blood, and proposed that production of Sly may correlate with increased severity of clinical symptoms and the capacity to reach higher colonization of organs. This notion is supported by the study of King *et al* (2001) [59] who reported that *sly* was present in a significantly higher number of isolates from pigs with meningitis, septicemia, and arthritis than isolates from pigs with pneumonia. Conversely, *sly* is also significantly overrepresented in asymptomatic carriage strains of pigs [59], showing that presence of *sly* does not exclusively correlate with invasive isolates. In addition to suilysin, it is likely that other virulence factors are relevant for the invasion of connective tissue, such as the hyaluronidase and heparinase II/III-like proteins. Note that the function of these putative virulence proteins still has to be demonstrated. In contrast to our results, *sly* expression was not increased in a recent study which compared gene expression of a wild-type strain with a Δ*ccpA* mutant grown to stationary phase [43]. The reason for these different findings may be related to the use of THB growth medium [43], a rich medium containing glucose, instead of the supplemented CM medium that we used, or different growth stages or different physiological states of the bacteria in the different media. Lastly, it is also possible that expression of *sly* is controlled by a CcpA-independent carbon catabolite repression mechanism although a *cre* site is present in the *sly* promoter region.

We propose that at mucosal surfaces the high ratios of starch to glucose promotes production of extracellular ApuA to support growth of *S. suis* in the oropharyngeal cavity. Additionally relief from CCR leads to higher expression of other virulence genes involved in mucosal infection (Figure 9). Indeed, in an *in vitro* experiment employing tracheal epithelial cells and *S. suis* bacteria grown with starch/pullulan or glucose as sole carbon source, a nearly 10-fold increase of epithelial cell invasion was observed when bacteria were grown with pullulan. We observed that in presence of starch, *S. suis* induced expression of the metabolic pathway enzymes required for transport and metabolism of maltodextrins and the glycogen biosynthesis enzymes which generate bacterial glycogen energy reserves. Several studies have linked bacterial glycogen metabolism to environmental survival, symbiotic performance, and colonization and virulence [60]–[67]. Glycogen reserves may be important for survival and fitness of *S. suis* when carbon sources are scarce. During colonization, relief from CCR would increase production of suilysin and may facilitate dispersion of *S. suis* into the deeper tissues due to loss of barrier integrity [4], [53], [58]. We hypothesized that once *S. suis* would reach the bloodstream, higher glucose concentrations (from 4.4 to 6.6 mM) would repress expression of *apuA* and *sly* by CCR (Figure 9). Indeed, gene expression analysis of bacteria isolated from the blood or organs of acutely infected pigs showed significantly lower expression of *apuA* and *sly* in blood than in the organs (Figure 8) supporting occurrence of CCR. In contrast, *S. suis* isolated from infected joints, heart or brain tissue expressed significantly higher levels of *sly* and *apuA* than bacteria isolated from blood. These results are probably due to the lower levels of glucose in the tissues (0.25–0.55 mM in normal synovial fluid and joint cavities) than in the blood (from 4.4 to 6.6 mM) [68], [69] and thus, relief from CCR when bacteria were located in the organs. In inflamed tissues, glucose levels may have been reduced further due to the consumption of glucose by neutrophils and macrophages. It is tempting to speculate that induction of suilysin expression in the tissues will release host glycogen from damaged cells that would be degraded by ApuA and metabolized to further support growth of *S. suis* (Figure 9). Additionally *S. suis* may be able to metabolize host glucans such as hyaluronan which is present in high concentrations (3–4 mg/ml) in synovial fluid [70].

Taken together, our results clearly demonstrate that the availability of glucose and other carbohydrates such as starch serves as an environmental cue to regulate the expression of *apuA* and other virulence genes. We propose a schematic model of how carbohydrate content of different tissues could modulate *S. suis* metabolism at the different stages of infection (Figure 9). Our novel findings demonstrate a regulatory mechanism dependent on relief from CcpA repression that links carbohydrate metabolism and virulence at least *in vitro* and that might play roles *in vivo*, at different stages of *S. suis* infection. Awareness of the role of carbohydrate content of the *S. suis* environment may lead to new strategies for combating this important disease, for instance via modulation of carbohydrate content and composition of animal feeds, or the inhibition of *S. suis* enzymes required for metabolism of starch.

## Supporting Information

Figure S1 *S. suis* S10 growth curve at 37°C in CM containing pullulan or glucose at 1% w/v.
(TIF)

Figure S2 GO term distribution of *S. suis* genes with the annotation “carbohydrate metabolism” that were differentially upregulated in starch/pullulan vs glucose. **A**. early exponential (e) and **B**. early stationary phase (s) Enrichment analysis performed using BLAST2GO (P = 0.05, two-tailed Fisher's Exact test).
(TIF)

Figure S3 Putative and characterized virulence gene expression ratios in pullulan vs. glucose. The genes are grouped according to their predicted or described function in *S. suis* pathogenesis and expression ratios are shown for exponential (white bar) and early stationary (black bar) growth phases. Envelope: cps2E*-SS0519 putative galactosyl transferase; wchF*-SSU0520 putative rhamnosyl transferase; *cps2C*-SSU0517 tyrosine-protein kinase; *cpss_lpl*-SSU1123 putative glycosyltransferase; *cpss_lpl2*-SSU1124 putative rhamnosyl transferase *pgdA*-SSU1448 peptidoglycan GlcNAc deacetylase, *dltA*-SSU0596 D-alanine-poly(phosphoribitol) ligase subunit1; Envelope/Adhesion *neuB*-SSU0535 putative N-acetylneuraminic acid synthase; *neuC*-SSU0536 putative UDP-N acetylglucosamine 2-epimerase; *neuA*-SSU0538 N-acylneuraminate cytidylyltransferase; Adhesion: apuA*-SSU1849 amylopullulanase; *sadP*-SSU0253 putative surface-anchored protein receptor; *gnd*-SSU1541 6-phosphogluconate dehydrogenase; Adhesion/Invasion: *srtA*-SSU0925 sortase; *fbpS*-SSU1311 fibronectin-fibrinogen binding protein; *gapdH*-SSU0153 glyceraldehyde-3-phosphate dehydrogenase; *eno*-SSU1320 enolase; *pepD*-SSU1215 putative surface-anchored dipeptidase; *dpp IV*-SSU0187 Xaa-Pro dipeptidyl-peptidase; Invasion: sly* SSU1231 suilysin (haemolysin); hepII/III*-SSU1048 heparinase II/III-like protein; hyl*-SSU1050 hyaluronidase precursor; ssnA*-SSU1760 surface-anchored DNA nuclease; arcB*-SSU0580 arginine deaminase; *igaP*-SSU1773 putative surface-anchored serine protease; *sspA*-SSU0757 cell envelope proteinase; *ofs*-SSU1474 serum opacity factor; *luxS*-SSU0376 S-ribosyl homocysteinase; Marker: *mrp*-SSU0706 muramidase-released protein precursor; *ef*-SSU0171 putative surface-anchored protein. * Indicates the presence of a predicted *cre* in the virulence gene promoter region.
(TIF)

Figure S4 Gene homologues in Gram-positive bacteria that share ≥45% of protein identity with the *S. suis apuR* gene (black arrows). The annotations of the genes downstream of *apuR* are also indicated and colored to show functional relatedness. Gene names are indicated above the arrows.
(TIF)

Figure S5 SDS polyacrylamide electrophoresis of purified transcriptional regulators. **A**. Coomassie stained SDS-PAGE gel (12%), showing purified fraction His-ApuR at the expected size of 38 kDa and **B**. Western Blot of the same gel with a monoclonal His-tag antibody protein. **C**. Coomassie stained SDS-PAGE gel (12%) of purified His-CcpA at expected size of 40 kDa.
(TIF)

Figure S6 EMSA with purified ApuA and CcpA. **A**. Schematic representation of the *apuA* promoter (P*_apuA_*) and fluorescently labeled PCR amplified DNA fragments (Pr1, Pr2 and Pr3) used for EMSAs. **B**. to **F**. DNA amplicons and DNA/rApuR protein complexes visualized in native 5% acrylamide gels using the Odyssey Imager. In these EMSA assays the concentration of each DNA amplicon was around 6 ng (∼50 nM) **B**. EMSA of 100 nM rApuR binding to Pr1, Pr2 and Pr3. **C**. Increasing DNA/rApuR complex formation in presence of 4 ng Pr2 DNA amplicon and an increasing amount of rApuR (∼0,5–4 µM as indicated) **D**. Competitive EMSA using increasing concentrations of non-fluorescent non-specific competitor DNA (lacking OM1 binding motifs). **E**. Competitive EMSA using increasing concentrations of non-fluorescent Pr2 as a specific competitor. The amounts of competitor DNA added are indicated (25–150 nM) **F**. Identification of specific ApuR binding sites in P*_apuA_*. PR2: native promoter region fragment 2. ΔOM1 and ΔOM2/*cre* are synthetic DNA fragments of P*_apuA_* that lack the predicted binding sites. + rApuR recombinant present - rApuR recombinant absent. **G**. to **J**. DNA amplicons and DNA/rCcpA protein complexes visualized in native 5% acrylamide gels using the Odyssey Imager. **G**. DNA/rCcpA complexes in the presence of increasing amounts of rCcpA as indicated. **H**. Competitive EMSA using increasing concentrations of non-fluorescent specific competitor **I**. Competitive EMSA using increasing concentrations of non-fluorescent non-specific competitor DNA (lacking OM2/cre binding motifs) **J**. DNA/rCcpA complex formation with fluorescent Pr2 and two synthetic promoter Pr2 fragments lacking either OM2/cre or OM1.
(TIF)

Table S1 Oligonucleotide primers used in this study.
(DOCX)

Table S2 Microarray data (xl file): *S. suis* genes differentially regulated during growth in pullulan (Pul) vs glucose (Glc) in the exponential (e) or stationary (s) phase.
(XLSX)

Table S3 *cre*-site prediction in the genome of *S. suis* P1/7.
(DOCX)

Table S4 Motif OM1-OM2/*cre* like motifs identified in *S. suis* 2 P1/7 and other Gram positive bacteria.
(DOCX)

Text S1 Material and methods.
(DOCX)

Text S2 Results (Supporting text on differentially expressed genes, pathways and their predicted or known functions).
(DOCX)
