# Supplementary figures and images for: Carbohydrate Availability Regulates Virulence Gene Expression in Streptococcus suis
Source: PLoS One. 2014 Mar 18;9(3):e89334. doi: 10.1371/journal.pone.0089334 (PMC3958366; doi:10.1371/journal.pone.0089334)

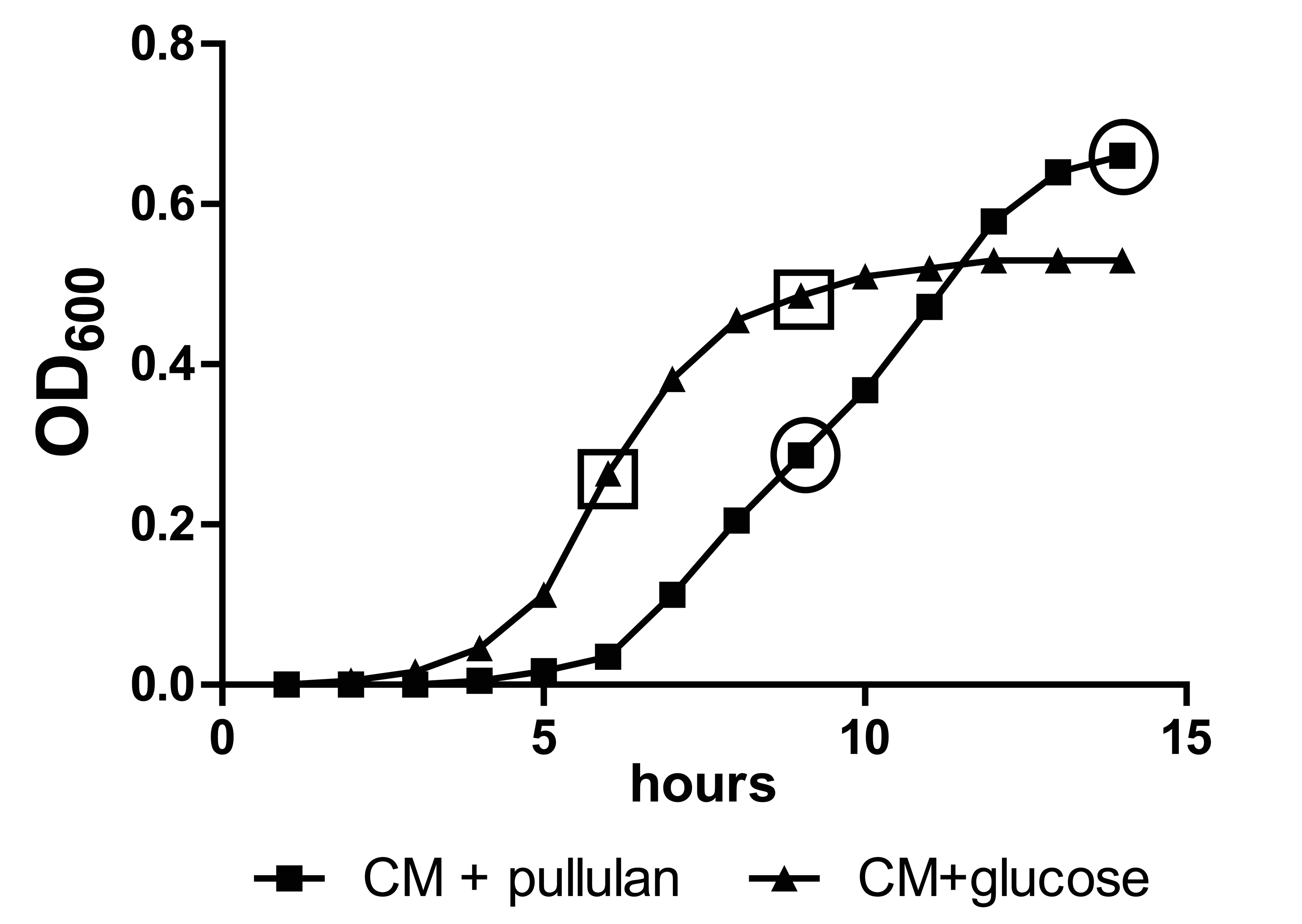

Supplement: Figure S1 — S. suis S10 growth curve at 37°C in CM containing pullulan or glucose at 1% w/v. (TIF) [file pone.0089334.s001.tif]

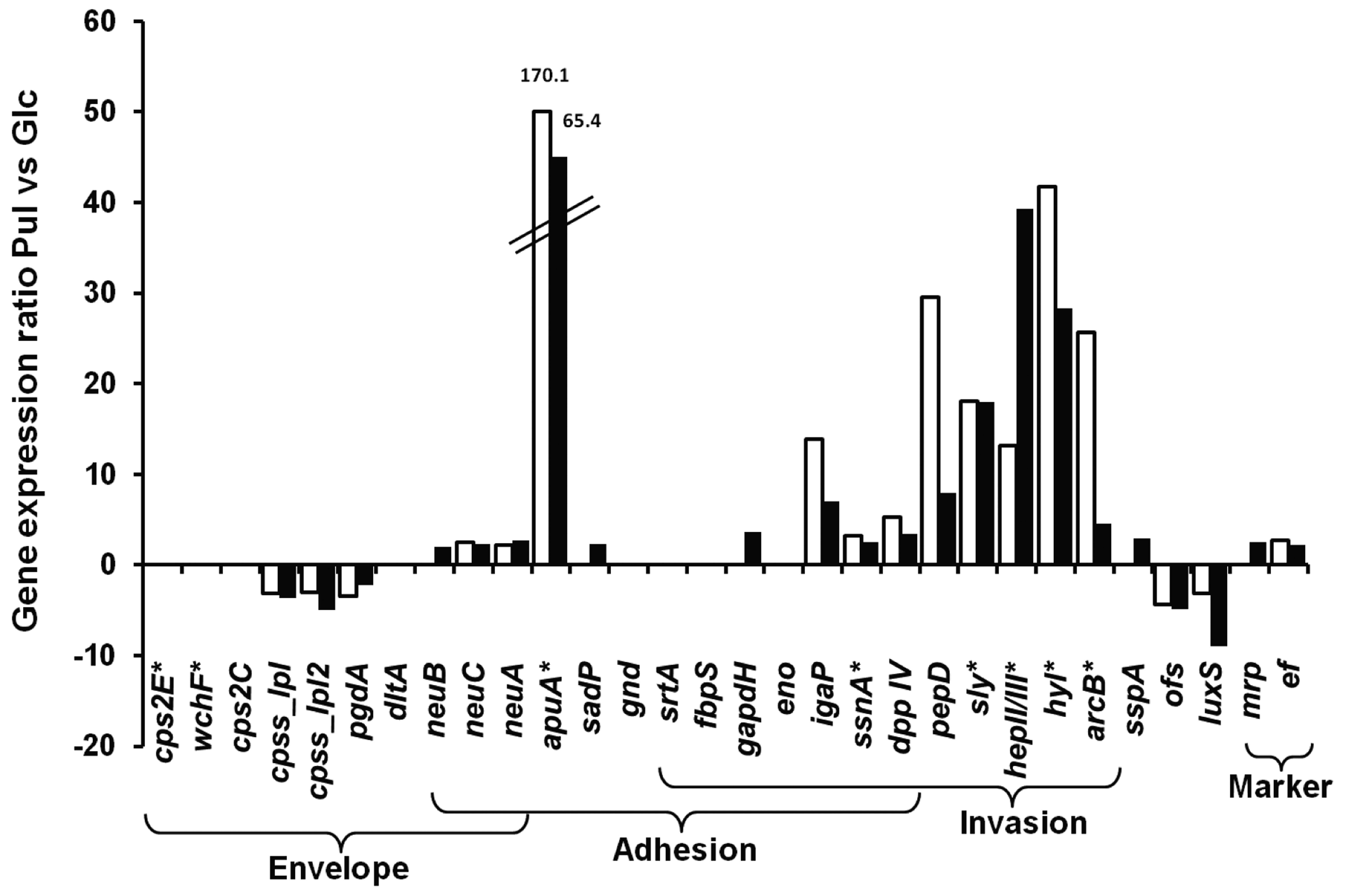

Supplement: Figure S3 — Putative and characterized virulence gene expression ratios in pullulan vs. glucose. The genes are grouped according to their predicted or described function in S. suis pathogenesis and expression ratios are shown for exponential (white bar) and early stationary (black bar) growth phases. Envelope: cps2E*-SS0519 putative galactosyl transferase; wchF*-SSU0520 putative rhamnosyl transferase; cps2C-SSU0517 tyrosine-protein kinase; cpss_lpl-SSU1123 putative glycosyltransferase; cpss_lpl2-SSU1124 putative rhamnosyl transferase pgdA-SSU1448 peptidoglycan GlcNAc deacetylase, dltA-SSU0596 D-alanine-poly(phosphoribitol) ligase subunit1; Envelope/Adhesion neuB-SSU0535 putative N-acetylneuraminic acid synthase; neuC-SSU0536 putative UDP-N acetylglucosamine 2-epimerase; neuA-SSU0538 N-acylneuraminate cytidylyltransferase; Adhesion: apuA*-SSU1849 amylopullulanase; sadP-SSU0253 putative surface-anchored protein receptor; gnd-SSU1541 6-phosphogluconate dehydrogenase; Adhesion/Invasion: srtA-SSU0925 sortase; fbpS-SSU1311 fibronectin-fibrinogen binding protein; gapdH-SSU0153 glyceraldehyde-3-phosphate dehydrogenase; eno-SSU1320 enolase; pepD-SSU1215 putative surface-anchored dipeptidase; dpp IV-SSU0187 Xaa-Pro dipeptidyl-peptidase; Invasion: sly* SSU1231 suilysin (haemolysin); hepII/III*-SSU1048 heparinase II/III-like protein; hyl*-SSU1050 hyaluronidase precursor; ssnA*-SSU1760 surface-anchored DNA nuclease; arcB*-SSU0580 arginine deaminase; igaP-SSU1773 putative surface-anchored serine protease; sspA-SSU0757 cell envelope proteinase; ofs-SSU1474 serum opacity factor; luxS-SSU0376 S-ribosyl homocysteinase; Marker: mrp-SSU0706 muramidase-released protein precursor; ef-SSU0171 putative surface-anchored protein. * Indicates the presence of a predicted cre in the virulence gene promoter region. (TIF) [file pone.0089334.s003.tif]

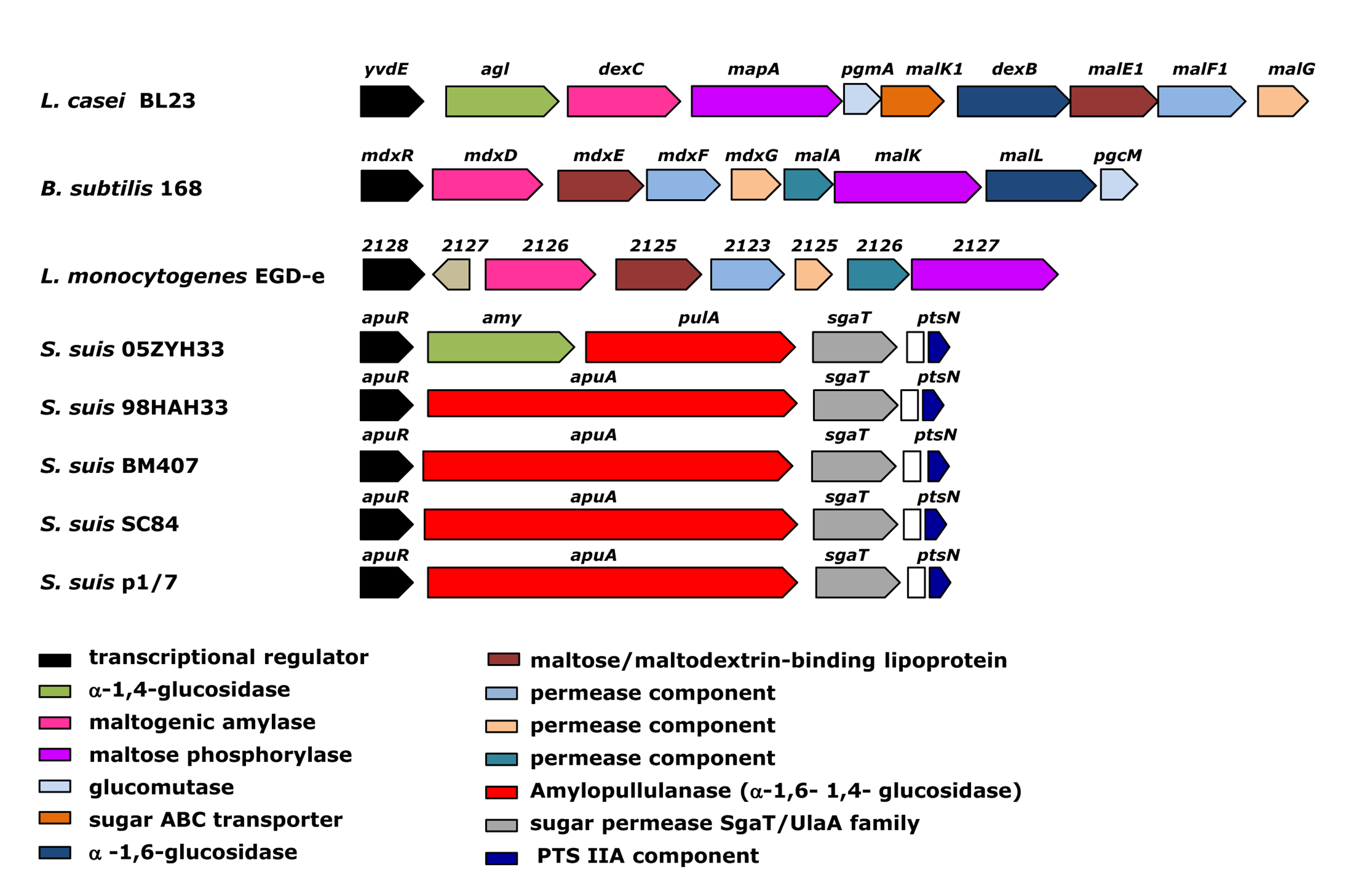

Supplement: Figure S4 — Gene homologues in Gram-positive bacteria that share ≥45% of protein identity with the S. suis apuR gene (black arrows). The annotations of the genes downstream of apuR are also indicated and colored to show functional relatedness. Gene names are indicated above the arrows. (TIF) [file pone.0089334.s004.tif]
