# Supplementary material for: Carbohydrate Availability Regulates Virulence Gene Expression in Streptococcus suis
Source: PLoS One. 2014 Mar 18;9(3):e89334. doi: 10.1371/journal.pone.0089334 (PMC3958366; doi:10.1371/journal.pone.0089334)
Supplement: Table S1 — Oligonucleotide primers used in this study. (DOCX) [file pone.0089334.s007.docx]

**Table S1** Oligonucleotide primers used in this study

| **Primer** | **Sequence^a^ (5′ to 3′)** | **Purpose** |
| --- | --- | --- |
| ApuR_F  ApuR_R | ACGACATTAGCCGATGTGG  CTGAGGTGTAGTCTCCCTTTCAA | To produce rApuR |
| CcpA_F  CcpA_R | TTAAACACTGACGATACGGTAACG  CTTAGTTGATTTACGTACTTTGATTCC | To produce rCcpA |
| Pr1F  Pr1R | AGGGAGACTACACCTCAGTA  TTAACGGTAACAAGTTTTGA | EMSA fragment Pr1 |
| Pr2F  Pr2R | AAAGAAGGGGGAGCTATTTAT  CTACCAGTATATACAATTCCAAGG | EMSA fragment Pr2 |
| Pr3F  Pr3R | CTTGTTACCGTTAACATTTAA  TATTCTGAACGGATTTCTT | EMSA fragment Pr3 |
| A-CompF  A-CompR | GTTTATCAAGGTGACTTCAGA  GTACCAATTCATCAAAGGA | Aspecific competitor |
| ΔOM1 | AAAGAAGGGGGAGCTATTTATTCTATTTTTGTTAGATAGTAGCTCAAAACT  TGTTACCGTTAACATTTAAACTCCTTGGAATTGTATATACTGGTAG | Synthetic fragment 1  with OM1 deletion |
| ΔOM2/*cre* | AAAGAAGGGGGAGCTATTTATTCTATTTTTGTTAGATAGTAGCTCAAAA  TAAGAAAACGTTTGCAAAGACTCCTTGGAATTGTATATACTGGTAG | Synthetic fragment 2  with OM2 deletion |
| ASP1  ASP2  AAP | CTCCCCAAGTCCAAA  CTGTTACCGCTGTCGCTTCA  GGCCACGCGTCGACTAGTACGGGIIGGGIIGGGIIG | 5*’*-RACE cDNA  5*’*-RACE nested PCR  5*’*-RACE nested PCR |
| proS1  proS2 | TTACGTGCGGGCTATGT  GCTGTAGCCGTCTTTCATG | qPCR *proS* reference gene |
| apuA1  apuA2 | CACCACTTGTCGCTTGTC  ACCTTTGACTGCAACAGTG | qPCR *apuA* target gene *in vitro* |
| apuA_Fw  apuA_Rev | ACTTCATGATGACAGACCGTTTCTAT  AAGTAGTCCAGCTTGGCTGTCACT | qPCR *apuA* target gene *in vivo* |
| Sly_fw  Sly_rev | CTGTTTATGTTTCCAGCGTTTCTTAT  GCCACTAATATCAACGCCTTTGA | qPCR sly target gene in vivo |
