# Supplementary material for: Carbohydrate Availability Regulates Virulence Gene Expression in Streptococcus suis
Source: PLoS One. 2014 Mar 18;9(3):e89334. doi: 10.1371/journal.pone.0089334 (PMC3958366; doi:10.1371/journal.pone.0089334)
Supplement: Table S3 — cre-site prediction in the genome of S. suis P1/7. (DOCX) [file pone.0089334.s009.docx]

**Table S3.** *cre*-site prediction in the genome of *S. suis* P1/7.

| **Sequence** | **P/Glc^1^** | **Locus** | **Operon *S. suis* P1/7** | **Pos^3^** | **COG** | **ΔccpA^2^** |
| --- | --- | --- | --- | --- | --- | --- |
| GAAAACGTTTGC | U | SSU1849 | *apuA* | P | Carbohydrate metab/virul |  |
| GAAAACGTTTGC | U | SSU1850 | *apuR* | P | Carbohydrate regulator |  |
| GAAAACGTTTGC | U | SSU1915 | *malX-malC-malD-malA-malR* | P | ABC Carbohydrate transport | U |
| GAAAACGTTTGC | U | SSU0844 | SSU0844 | P | Unknown function | U |
| GAAAACGTTTGC | U | SSU1914 | *malQ1* | P | Carbohydrate metabolism |  |
| GCAAACGTTTGC | U | SSU0357 | *ptsG-elsH* | P | PTS Carbohydrate transport |  |
| GAATACGTTCGC | U | SSU0766 | *glpR-fruB-fruA* | G | PTS Carbohydrate transport | U |
| GATAATGTTTGC | U | SSU1583 | *manL-manlM-manN* | G | PTS Carbohydrate transport | U |
| GAACGCGTTTGC | D | SSU1320 | *eno* | G | Carbohydrate metabolism | D |
| GAAAACGTCGGC | D | SSU1467 | *xseA-xseB-fps-SSU1464-argR-recN* | P | DNA replication |  |
| GAAAACGTTTAC | D | SSU0249 | *gla* | P | Energy production | D |
| GAAAACGTTTAC | D | SSU1256 | *dnaE-rpoD* | P | Transcription |  |
| GAAAACGGTTGC | U | SSU0395 | SSU0395 | P | Unknown | U |
| GTAAACGTTTGC | D | SSU0355 | *gntR* | P | Carbohydrate regulator |  |
| GTAAACGTTTGC | U | SSU1726 | *murQ-ptsG-*SSU1724 | P | Carbohydrate transp/metab |  |
| GTAAGCGGTTGC | U | SSU0402 | *lacZ-*SSU0403*-manY-manZ-manX* | P | Carbohydrate metabolism |  |
| GGAAGCGGTTGC | U | SSU1050 | *hyl-hepII/III* | P | Virulence | U |
| TAAAACGTTTAC |  | SSU0245 | SSU0245-SSU0246-SSU0247 | P | Regulation multidrug efflux |  |
| GAAAACGTTTTG | D | SSU0284 | *his-hisM* | P | Amino acid transporter | D |
| AAAAACGTTTTC | D | SSU0353 | *malQ2-glgP2* | P | Carbohydrate metabolism | D |
| GAAAACATTTAC | D | SSU0421 | *typA-*SSU0422 | P | Energy production | D |
| AAAAACGTTTGG |  | SSU0449 | *SSU0449-SSU0450-SSU0451* | P | Unknown |  |
| AAAAACGTTTGG |  | SSU0524 | *csp2H* | P | Envelope/virulence | D |
| AAAAACGTTTTC | U | SSU0938 | *rsmC-deoA-deoC-cdd* | P | Energy production | U |
| AAAAACGTTTTC |  | SSU0939 | *coaA* | P | Energy production | U |
| GAAAACATTTGG | U | SSU1688 | *paaD* | P | Unknown |  |
| GAAAACTTTTGT | D | SSU1969 | *HtrA-Spo0J* | P | DNA replication |  |
| GAAAACGATTAC | U | SSU0328 | *galR-galK-galT* | P | Carbohydrate regulator | U |
| GAAAACGATTAC | U | SSU0329 | *galK-galT* | P | Carbohydrate metabolism | U |
| GAAGAAGTTTGC | D | SSU0375 | *SSU0375-SSU0374* | P | Unknown |  |
| GAAAACGGTTAC | D | SSU0377 | *SSU0377* | P | Hydrolase |  |
| GATAACGTTTTC | D | SSU0515 | *cps2A* | P | Envelope/virulence | D |
| GAAATCGTTTTC |  | SSU0613 | SSU0613 | P | Carbohydrate regulator | U |
| GTAAATGTTTGC | U | SSU1033 | SSU1033 | P | Energy production |  |
| GAAAACGGTTAC | U | SSU1172 | SSU1172-SSU1171-SSU1170-*invrtsC* | P | Carbohydrate transp/metab. | U |
| GAAAACGGTTAC | U | SSU1175 | SSU1175 | P | Unknown | U |
| GTAAACGTTTTC | U | SSU1265 | *glgP1* | P | Carbohydrate metabolism | U |
| GTAAACGTTTTC |  | SSU1618 | *scrA-ScrK* | P | PTS Carbohydrate transport |  |
| GTAAACGTTTTC |  | SSU1619 | *scrB-scrR* | P | Carbohydrate metabolism |  |
| GAAAAGGTTAGC | D | SSU1794 | *stdB*-SSU1795-SSU1794*-ksgA* | P | Energy production |  |
| GATAACGTTTGA |  | SSU1883 | *srtB-srtC-srtD* | P | Hydrolase |  |
| GAAAAAGCTTGC | D | SSU0006 | SSU0006*-pth-trcF-*SSU0009-SSU0010-SSU0011*-penP-mesJ-hpt-ftsH* | P | Energy production | D |
| GCAAACGTTTTC | U | SSU0240 | *SSU0240-SSU0241-SSU0242* | P | Unknown |  |
| AAAAACGCTTGC | D | SSU0287 | *SSU0287-SSU0288* | P | Unknown | D |
| GCAAACGTTTAC | U | SSU0356 | *elsH-ptsG* | P | Unknown |  |
| GAAAATGCTTGC | U | SSU0482 | *tufA* | P | DNA replication |  |
| GAAAAGGCTTGC | U | SSU0673 | *hcaD-SSU0674-glpK-glpO-glpF1* | P | Carbohydrate metabolism | U |
| GCAAAGGTTTGC | U | SSU0775 | *glkA* | P | Carbohydrate transport |  |
| GACAACGTTTAC | D | SSu0789 | SSU0789*-tdK* | P | Unknown | D |
| GAAAATGTTCGC | D | SSU1960 | *guaA* | P | Energy production |  |
| GAAAGCGTTTCC | U | SSU0870 | *glgC-glgC-glgA-glgB* | P | Carbohydrate metabolism | U |
| GAAAACGCTTAC | U | SSU1008 | *ptsMelB-pfkB-uidA-fadR-dgoA-uxaC-kduD-Had-bglX-bglX* | P | Carbohydrate transport | U |
| GCAAAGGTTTGC | D | SSU1183 | SSU1183 | P | Stress response |  |
| ACAAACGTTTGC | U | SSU1231 | *sly* | P | Virulence |  |
| GAAAACGCTTTC | U | SSU1713 | SSU1713*-ams-nag-gcnA* | P | Carbohydrate metabolism | U |
| AAAAACGTCTGC | U | SSU1760 | *ssnA* | P | Virulence |  |
| TAAAGCGTTTTCA | U | SSU1373 | *aga-msmE-msmF* | P | Carbohydrate metabolism |  |
| GAAAACGCTTCC | U | SSU0260 | *adhE-adhP* | P | Energy production | U |
| GAAAACGCTTCC | U | SSU0675 | *glpK-glpO-glpF1* | P | Energy production | U |
| GAAATGCTTGTCG | D | SSU1308 | *bglB-ptsG-*SSU1310 | P | Carbohydrate metabolism | D |
| GAGAACGTATGC |  | SSU0558 | *aroK-pheA-LytR* | P | Amino acid transport |  |
| CAAAACTTTTGC |  | SSU0134 | *ackA* | G | Carbohydrate metabolism | U |
| GAAACCGCTTCGA | U | SSU0856 | *pgm* | P | Carbohydrate metabolism | U |
| GTAAACGGTTGC | U | SSU0766 | *glpR-fruB-fruA* | P | Carbohydrate regulator | U |
| TAAAGCGTTTTCA | U | SSU1701 | *msmK* | P | ABC Carbohydrate transport | U |
| GAAAGGTTTTTCA | U | SSU0191 | *pflB* | P | Energy production | U |
| CAAAGGAGTTTT | U | SSU1749 | *dak1-dak1-SSU1751-glpF2* | P | Energy production | D |
| GAAAACGATGGC | U | SSU0580 | *arcA-arcB-arcC* | G | Energy produc/virulence | U |
| GAAAACGATGGC | U | SSU0347 | SSU0347-SSU0348-SSU0349-SSU0350*-fms* | G | Energy production | U |
| GAAAACGACTTC | U | SSU1855 | *ptsCelB* | P | PTS Carbohydrate transport | U |
| TGAAACCACTTT | U | SSU1203 | *pepQ* | P | Virulence |  |
| TGAAAGCGCATAA | U | SSU0187 | *dppIV* | P | Virulence |  |

Δ*ccpA*^2^ upregulated (U) or downregulated (D) expression in wild type *S. suis* 10 compared to its isogenic *∆ccpA* mutant (data from Willenborg *et al*. [44])

Pos^3^ tag locus located upstream of the start codon of predicted proteins (P) or in proximity of the gene transcription start (G)
