# Supplementary material for: Carbohydrate Availability Regulates Virulence Gene Expression in Streptococcus suis
Source: PLoS One. 2014 Mar 18;9(3):e89334. doi: 10.1371/journal.pone.0089334 (PMC3958366; doi:10.1371/journal.pone.0089334)
Supplement: Table S4 — Motif OM1-OM2/cre like motifs identified in S. suis 2 P1/7 and other Gram positive bacteria. (DOCX) [file pone.0089334.s010.docx]

**Table S4.** Motif OM1-OM2/*cre* like motifs identified in *S. suis* 2 P1/7 and other Gram positive bacteria.

| **Strain** | **Gene** | **Position^a^** | **Regulator** | ***mdxR site*** | **Locus tag** | **reference** |
| --- | --- | --- | --- | --- | --- | --- |
| *S. suis* | *apuA* | -82 | ApuR | TTGTTACCGtTAACA | SSU1849 | this work |
| *S. suis* | *malQ* | -59 | ApuR | TTGTTACCGtTttCA | SSU1914 | predicted |
| *S. suis* | *malP* | -181 | ApuR | ccGTaACCGtTttCt | SSU0357 | predicted |
| *B. subtilis str. 168* | *mdxE* | -74 | MdxR | TTGTTACCGgTAACA | [BSU34610](http://www.microbesonline.org/cgi-bin/fetchLocus.cgi?locus=40000&disp=0) | RegPrecise |
| *L. monocytogenes* EGD-e | *lmo2125* | -152 | Lmo2128 | cTGTTACCGgTAACA | Lmo2125 | RegPrecise |
| **Strain** | **Gene** | **Position^a^** | **Regulator** | ***cre site*** | **Locus tag** | **reference** |
| *S. suis* | *apuA* | -61 | CcpA | TGcAAaCGtTttCtT | SSU1849 | this work |
| *S. suis* | *malX* | -43 | CcpA | cGcAAaCGtTttCcT | SSU1915 | predicted |
| *S. suis* | *malQ1* | -26 | CcpA | aGAAAaCGtTtgCgT | SSU1914 | predicted |
| *S. suis* | *apuR* | -60 | CcpA | TGcAAaCGtTttCtT | SSU1850 | predicted |
| *S. suis* | *malP* | -149 | CcpA | aGcAAaCGtTtgCAT | SSU0357 | predicted |
| *B. subtilis str. 168* | *mdxE* | -49 | CcpA | TGTAAcCGcTttCAT | [BSU34610](http://www.microbesonline.org/cgi-bin/fetchLocus.cgi?locus=40000&disp=0) | predicted |
| *L. monocytogenes* EGD-e | *Lmo2125* | -126 | CcpA | TGTAAaCGcTttCgA | Lmo2125 | predicted |
| *S. sanguinis SK36* | *pulA* | -57 | CcpA | aGAAAaCGtTtgCAT | [SSA_0453](http://www.microbesonline.org/cgi-bin/fetchLocus.cgi?locus=2060027&disp=0) | RegPrecise |
| *S. sanguinis SK36* | *pulA* | -100 | CcpA | TGAAAgCctTtaCAA | [SSA_0453](http://www.microbesonline.org/cgi-bin/fetchLocus.cgi?locus=2060027&disp=0) | RegPrecise |
| *S. pyogenes M1 GAS* | *pulA* | -65 | CcpA | TGcAAgCGcTtgCgc | [SPy_1972](http://www.microbesonline.org/cgi-bin/fetchLocus.cgi?locus=102084&disp=0) | RegPrecise |
| *S. pneumoniae TIGR4* | *pulA* | -69 | CcpA | aGAAAaCGcTtgCAT | [SP_0268](http://www.microbesonline.org/cgi-bin/fetchLocus.cgi?locus=115805&disp=0) | RegPrecise |
| *S. agalactiae 2603V/R* | *pulA* | -81 | CcpA | aGAAAtCatTtaCtT | [SAG1216](http://www.microbesonline.org/cgi-bin/fetchLocus.cgi?locus=275846&disp=0) | RegPrecise |
| *S. equi MGCS10565* | *pulA* | -76 | CcpA | aGAAAaCGaTtgCAc | [Sez_0820](http://www.microbesonline.org/cgi-bin/fetchLocus.cgi?locus=5804347&disp=0) | RegPrecise |

^a^ Position relative to translation starting codon.

^b^ *Bacillus sp*. consensus MdxR NTGWWWCCGNTWNCA (N=any base; W=A or T)

^c^ *Bacillus sp*. consensus *cre* TGWAANCGNTNWCAW (N=any base; W=A or T)
