# Supplementary material for: Carbohydrate Availability Regulates Virulence Gene Expression in Streptococcus suis
Source: PLoS One. 2014 Mar 18;9(3):e89334. doi: 10.1371/journal.pone.0089334 (PMC3958366; doi:10.1371/journal.pone.0089334)
Supplement: Text S1 — Material and methods. (DOCX) [file pone.0089334.s011.docx]

**Text S1 material and methods:**

***Growing bacteria in complex media***

A complex medium (CM) comprised 10 g/l proteose peptone, 5 g/l trypticase peptone, 5 g/l yeast extract, 2.5 g/l KCl, 1 mM Urea, and 1 mM Arginine, pH 7.0 was used to assess growth on different carbon sources by supplementation with different carbohydrates (glucose: Glc, pullulan: Pul, lactose: Lact, maltotriose: Maltr) at final concentrations of 1% (wt/vol).

***Statistics qPCR***

Constitutive gene expression in complex media was determined as a ratio of target gene vs reference gene *proS* and was calculated according to the following equation: ratio = (*E*_reference_)^Ct^ ^reference^/(*E*_target_)^Ct target^ where *E* is the amplification efficiency and Ct is the number of PCR cycles needed for the signal to exceed a specific threshold value.

***Recombinant regulators production and Infrared EMSA***

The recombinant ApuR (rApuR) and CcpA (rCcpA) proteins were cloned in pTrcHis TOPO2 TA. The purified PCR products were ligated to the pTrcHis TOPO2 TA expression vector (Invitrogen). The expressed recombinant regulators were fused to a C-terminal polypeptide containing six histidine residues for affinity purification. After transformation of *E. coli* TOPO 10, the clones containing the recombinant ApuR (rApuR) and CcpA (rCcpA) were selected on LB agar containing 50 µg/ml of ampicillin; several colonies were checked for the correct insertion of the *apuR* and *ccpA* gene fragments and verified by DNA sequencing. Expression of both regulators was induced by addition of 1 mM isopropyl-β-D-1-thiogalattopiranoside (IPTG, Invitrogen) to an exponentially growing culture (OD_600_ of 0.6) for 4 hours at 18°C for ApuR and overnight at 18°C for CcpA under shaking (250 rpm). The cells were harvested by centrifugation (8000 g x 10 min at 4°C) and the pellet was suspended in lysis buffer (50 mM Tris-Cl; 0.5 M NaCl, pH 7.4) containing a cocktail of protease inhibitors (Roche), and then disrupted using a high pressure cell disrupter (Constant Systems, U.K.). The soluble protein extracts were recovered after high speed centrifugation (14000 rpm 40 min at 4°C) and purified by HPLC affinity chromatography (HisTrap affinity column, Amersham Pharmacia Biotech, Freiburg, Germany) using extensive washing of the column bound protein with a buffer containing low concentrations of imidazole to remove weakly binding proteins. His-tagged rApuR and rCcpA were then eluted using an imidazole gradient; fractions containing the highest purity were obtained using an imidazole concentration of around 100 mM. Fractions containing purified fusion proteins of the expected size (approx. 38 kDa for ApuR and 40 kDa for CcpA) were collected and dialyzed against buffer (500 mM NaCl, 50 mM Tris-HCl, pH 7.4) and stored at -80°C with 10% of glycerol. Protein concentrations were measured using a BCA Protein Assay (Thermo Scientific), according to the supplier's instructions.
