# Supplementary material for: Carbohydrate Availability Regulates Virulence Gene Expression in Streptococcus suis
Source: PLoS One. 2014 Mar 18;9(3):e89334. doi: 10.1371/journal.pone.0089334 (PMC3958366; doi:10.1371/journal.pone.0089334)
Supplement: Text S2 — Results (Supporting text on differentially expressed genes, pathways and their predicted or known functions). (DOCX) [file pone.0089334.s012.docx]

**Text S2**

**Results** (**Supporting text on differentially expressed genes, pathways and their predicted or known functions)**

***The effect of pullulan and glucose on carbohydrate metabolic pathways***

When *S. suis* was grown in pullulan, 12 out of the 17 annotated sugar phosphotransferase systems (PTS) genes were upregulated compared to bacteria grown in glucose as shown in other streptococci [[1](#_ENREF_1),[2](#_ENREF_2)] (Table S2). Only *ptsG-*IIBCA^Glc^ (SSU1309), a predicted glucose transporter was down-regulated (Table S2). Two PTS strongly upregulated in pullulan compared to glucose (i.e. SSU0404-5 and SSU1847) have high similarity to PTS that in other streptococci transport maltodextrins, the catalytic degradation products of pullulan generated by the cell-wall associated amylopullulanase ApuA. Additionally homologues of the maltodextrin-specific ATP-binding cassette (ABC) (*malX*, *malC* and *malD;* SSU1915, SSU1916, SSU1917) and maltose PTS *malT* (*ptsG*-SSU0357) found in *S. pneumoniae* TIGR4 SP_SP0758) [[1](#_ENREF_1),[3-8](#_ENREF_3)] were upregulated during growth in pullulan.

Uptake of maltose by a PTS generates maltose-6-P which is then cleaved into glucose-6-P and glucose by *MalA* [[9](#_ENREF_9)]. Maltodextrins transported into the cell are converted to glucose-1-phosphate by the action of maltodextrin utilization proteins such as α-1,4-glucosidase [[9](#_ENREF_9),[10](#_ENREF_10)], and maltodextrin phosphorylase [[9](#_ENREF_9),[11-14](#_ENREF_11)]. The predicted *S. suis* α-1,4-glucosidase (*malA*-SSU1918 or *malA1*-SSU1862), and maltodextrin phosphorylase (*glgP*1-SSU1265), genes were upregulated in pullulan versus glucose, suggesting that the pathway for conversion of maltodextrins to the phosphorylated form of α-glucose (α-glc), is also induced and conserved in *S. suis* (Figure 2A and B). Furthermore, two genes encoding putative transcriptional regulators (*apuR*-SSU1850 and *malR-*SSU1919) showed differential expression in pullulan (see further studies below).

Glucose-1-P (glc1P), released upon pullulan degradation, can be metabolized in different pathways. Phosphoglucomutase (*pgm-*SSU0826) which can isomerise glc1P to glc6P for entry into the glycolysis pathway was highly upregulated in pullulan compared to glucose [[9](#_ENREF_9)]. However, the glycolysis pathway enzymes were either not modulated or slightly down-regulated in pullulan compared to glucose in different growth phases (Figure 2). This is likely due to the fact that glycolysis is maximally expressed during growth in glucose as the sole carbon source. The pentose-glucuronate pathway genes were more highly transcribed in pullulan compared to glucose suggesting this alternative but less efficient pathway for energy production was used to metabolize accumulating glc6P that was not immediately metabolized by the glycolytic pathway (Figure 2A and B). Glc1P can be converted to glc6P for entry into glycolysis or pentose-glucuronate pathway but may also be used for the synthesis of glycogen as an energy reserve when an excess of carbon source is available. Indeed, glycogen biosynthesis genes were strongly induced in pullulan compared to glucose (*glgC-glgA-glgB*; SSU0870 to SSU0874) while the glycogen utilization genes (*glgP2-*SSU0354 and *malQ2* SSU0353) were not differentially expressed (Figure 2A and 2B).

In the genome of *S. suis* we found genes predicted to encode enzymes belonging to the starch-sucrose and galactose metabolism pathways that participate in the interconversion of sugars [[15](#_ENREF_15),[16](#_ENREF_16)]. Of these a predicted sucrose phosphorylase (*gtfA*-SSU1369), β-fructofuranosidase/interconvertase [[17](#_ENREF_17)] (*invrtsC*-SSU1169) and raffinose galactohydrolase [[18](#_ENREF_18)] (*rafgH*-SSU0167) were strongly induced in pullulan compared to glucose (Figure 2A and B) and may be associated with the downstream induction of genes associated with galactose metabolism (Figure 2, Table S2).

Interestingly, the *galT* (galactose-1-phosphate uridylyltransferase) and *galK* (galactokinase) genes that participate in the Leloir pathway were strongly induced in pullulan compared to glucose. The GalT enzyme (EC 2.7.7.9) interconverts galactose-1-phosphate (gal1-P) and UDP-Glucose (UDP-glc) to UDP-galactose (UDP-gal) and glc1P. Activated UDP-sugars are important intermediates in polysaccharide biosynthesis, e.g. as capsule components or exopolysaccharides. However, the expression ratios of capsule biosynthesis genes (*cps2C*-SSU0517, *cps2D*-SSU0518, *cps2E*-SSU0519, *cpss_lpl*-SS0520) were slightly lower for bacteria grown in pullulan compared to glucose. Additionally, capsule production by *S. suis* grown in pullulan and glucose appeared not to be different as judged by transmission electron micrographs (data no shown). *CcpA* (SSU1202) the global regulator of catabolite metabolism was upregulated during growth of *S. suis* in glucose compared to growth in pullulan (Table S2).

***S. suis CPS biosynthesis appears to be differentially regulated during growth in pullulan or glucose***

Expression of *galT* and *galK* which catalyze the interconversion of UDP-galactose (UDP-gal) to UDP-glucose (UDP-glc) [[19](#_ENREF_19),[20](#_ENREF_20)] were upregulated in pullulan compared to glucose (Figure 2). Activated sugars such as UDP-glc and UDP-gal are key components in the biosynthetic pathway of the *S. suis* capsule suggesting that their increased expression might have led to a corresponding increase in capsule production [[21-24](#_ENREF_21)]. However, we observed a slightly decreased expression of the capsule operon regulator (*cps2A*/*wzg*) and two glycosyl transferases (*cpss_lpl* and *cpss_lpl2)* in the capsule operon during growth in pullulan. Although no apparent difference in the thickness of the capsule was observed in electron transmission micrographs of *S. suis* grown in glucose or pullulan (data no shown) we cannot rule out reduced capsule production or altered composition during growth in pullulan. Reduced capsule production was observed in a recent study, where a *S. suis* S10 Δ*ccpA* mutant showed lower expression of the capsule compared to the wild type, in rich medium containing glucose [[25](#_ENREF_25)]. We also found a *cre* site in the promoter of the *cps* genes (Table S3) suggesting transcriptional regulation by CcpA. However, in some *Streptococcus* species, production of capsule is influenced by carbon source in a CcpA-independent manner [[26](#_ENREF_26),[27](#_ENREF_27)].

***Potential roles of putative virulence factors***

Two peptidyl peptidases (*dppIV* and *pepD*) were induced more strongly when *S. suis* was grown in pullulan. In *S. suis*, the function of *pepD* is unknown but the *S. suis* di-peptidyl peptidase IV (DppIV) has been shown to interact with human fibronectin, and a *dppIV*-deficient mutant was greatly attenuated in a mouse infection model [[28](#_ENREF_28)]. A putative heparinase II/III enzyme was also highly upregulated but its function and potential role in virulence is unknown. In our experiments, the *sspA* gene which encodes a secreted serine protease was also induced. This protease can degrade interleukin-8 (IL-8), known for its role in chemotaxis and recruitment of neutrophils from the vasculature to sites of infection or tissue injury *in vivo* [[29](#_ENREF_29),[30](#_ENREF_30)]. The *ssnA* gene, encoding a recently described DNase which is secreted by *S. suis* and potentially involved in the breakdown of neutrophil entrapments (NETS) [[31](#_ENREF_31)], was slightly upregulated in pullulan compared to glucose.

Several adhesion factors, namely ApuA [[32](#_ENREF_32)], FbpS [[33](#_ENREF_33)], Eno [[34](#_ENREF_34)], GAPDH [[35](#_ENREF_35),[36](#_ENREF_36)], Gnd [[37](#_ENREF_37)] and SadP [[38](#_ENREF_38)] have been characterized in *S. suis*. Transcription of *apuA* was strongly induced, whereas *sadP* and *gapdH* were only slightly induced during growth in pullulan compared to glucose. As predicted by the transcriptomics data we showed higher levels of adhesion and invasion of porcine epithelial cells when *S. suis* was grown on pullulan rather than glucose.

***SI Reference***

1. Bidossi A, Mulas L, Decorosi F, Colomba L, Ricci S, et al. (2012) A functional genomics approach to establish the complement of carbohydrate transporters in Streptococcus pneumoniae. PLoS One 7: e33320.

2. Deutscher J, Francke C, Postma PW (2006) How phosphotransferase system-related protein phosphorylation regulates carbohydrate metabolism in bacteria. Microbiol Mol Biol Rev 70: 939-1031.

3. Puyet A, Espinosa M (1993) Structure of the maltodextrin-uptake locus of Streptococcus pneumoniae. Correlation to the Escherichia coli maltose regulon. J Mol Biol 230: 800-811.

4. Shelburne SA, 3rd, Fang H, Okorafor N, Sumby P, Sitkiewicz I, et al. (2007) MalE of group A Streptococcus participates in the rapid transport of maltotriose and longer maltodextrins. J Bacteriol 189: 2610-2617.

5. Abbott DW, Higgins MA, Hyrnuik S, Pluvinage B, Lammerts van Bueren A, et al. (2010) The molecular basis of glycogen breakdown and transport in Streptococcus pneumoniae. Molecular microbiology 77: 183-199.

6. Abbott DW, Higgins MA, Hyrnuik S, Pluvinage B, Lammerts van Bueren A, et al. (2010) The molecular basis of glycogen breakdown and transport in Streptococcus pneumoniae. Mol Microbiol 77: 183-199.

7. Ajdic D, Pham VT (2007) Global transcriptional analysis of Streptococcus mutans sugar transporters using microarrays. J Bacteriol 189: 5049-5059.

8. Shelburne SA, 3rd, Keith DB, Davenport MT, Horstmann N, Brennan RG, et al. (2008) Molecular characterization of group A Streptococcus maltodextrin catabolism and its role in pharyngitis. Mol Microbiol 69: 436-452.

9. Schonert S, Seitz S, Krafft H, Feuerbaum EA, Andernach I, et al. (2006) Maltose and maltodextrin utilization by Bacillus subtilis. Journal of bacteriology 188: 3911-3922.

10. Nahalka J (2008) Physiological aggregation of maltodextrin phosphorylase from Pyrococcus furiosus and its application in a process of batch starch degradation to alpha-D-glucose-1-phosphate. J Ind Microbiol Biotechnol 35: 219-223.

11. Boos W, Shuman H (1998) Maltose/maltodextrin system of Escherichia coli: transport, metabolism, and regulation. Microbiol Mol Biol Rev 62: 204-229.

12. Jeon BS, Taguchi H, Sakai H, Ohshima T, Wakagi T, et al. (1997) 4-alpha-glucanotransferase from the hyperthermophilic archaeon Thermococcus litoralis--enzyme purification and characterization, and gene cloning, sequencing and expression in Escherichia coli. Eur J Biochem 248: 171-178.

13. Lee HS, Shockley KR, Schut GJ, Conners SB, Montero CI, et al. (2006) Transcriptional and biochemical analysis of starch metabolism in the hyperthermophilic archaeon Pyrococcus furiosus. J Bacteriol 188: 2115-2125.

14. Le Breton Y, Pichereau V, Sauvageot N, Auffray Y, Rince A (2005) Maltose utilization in Enterococcus faecalis. J Appl Microbiol 98: 806-813.

15. Taegtmeyer H (1985) Carbohydrate interconversions and energy production. Circulation 72: IV1-8.

16. Loewus FA (1971) Carbohydrate Interconversions. Ann Rev Plant Physiol 22: 337-364.

17. Ehrmann MA, Korakli M, Vogel RF (2003) Identification of the gene for beta-fructofuranosidase of Bifidobacterium lactis DSM10140(T) and characterization of the enzyme expressed in Escherichia coli. Curr Microbiol 46: 391-397.

18. Aduse-Opoku J, Tao L, Ferretti JJ, Russell RR (1991) Biochemical and genetic analysis of Streptococcus mutans alpha-galactosidase. J Gen Microbiol 137: 2271-2272.

19. Frey PA (1996) The Leloir pathway: a mechanistic imperative for three enzymes to change the stereochemical configuration of a single carbon in galactose. FASEB J 10: 461-470.

20. Holden HM, Rayment I, Thoden JB (2003) Structure and function of enzymes of the Leloir pathway for galactose metabolism. J Biol Chem 278: 43885-43888.

21. Van Calsteren MR, Gagnon F, Lacouture S, Fittipaldi N, Gottschalk M (2010) Structure determination of Streptococcus suis serotype 2 capsular polysaccharide. Biochem Cell Biol 88: 513-525.

22. Bar-Peled M, Griffith CL, Ory JJ, Doering TL (2004) Biosynthesis of UDP-GlcA, a key metabolite for capsular polysaccharide synthesis in the pathogenic fungus Cryptococcus neoformans. Biochem J 381: 131-136.

23. Mollerach M, Lopez R, Garcia E (1998) Characterization of the galU gene of Streptococcus pneumoniae encoding a uridine diphosphoglucose pyrophosphorylase: a gene essential for capsular polysaccharide biosynthesis. J Exp Med 188: 2047-2056.

24. Levander F, Svensson M, Radstrom P (2002) Enhanced exopolysaccharide production by metabolic engineering of Streptococcus thermophilus. Appl Environ Microbiol 68: 784-790.

25. Willenborg J, Fulde M, de Greeff A, Rohde M, Smith HE, et al. (2011) Role of glucose and CcpA in capsule expression and virulence of Streptococcus suis. Microbiology 157: 1823-1833.

26. Carvalho SM, Kloosterman TG, Kuipers OP, Neves AR (2011) CcpA ensures optimal metabolic fitness of Streptococcus pneumoniae. PLoS One 6: e26707.

27. Iyer R, Baliga NS, Camilli A (2005) Catabolite control protein A (CcpA) contributes to virulence and regulation of sugar metabolism in Streptococcus pneumoniae. J Bacteriol 187: 8340-8349.

28. Ge J, Feng Y, Ji H, Zhang H, Zheng F, et al. (2009) Inactivation of dipeptidyl peptidase IV attenuates the virulence of Streptococcus suis serotype 2 that causes streptococcal toxic shock syndrome. Curr Microbiol 59: 248-255.

29. Bonifait L, Vaillancourt K, Gottschalk M, Frenette M, Grenier D (2010) Purification and characterization of the subtilisin-like protease of Streptococcus suis that contributes to its virulence. Vet Microbiol 148: 333-340.

30. Bonifait L, Grenier D (2011) The SspA subtilisin-like protease of Streptococcus suis triggers a pro-inflammatory response in macrophages through a non-proteolytic mechanism. BMC Microbiol 11: 47.

31. Buchanan JT, Simpson AJ, Aziz RK, Liu GY, Kristian SA, et al. (2006) DNase expression allows the pathogen group A Streptococcus to escape killing in neutrophil extracellular traps. Curr Biol 16: 396-400.

32. Ferrando ML, Fuentes S, de Greeff A, Smith H, Wells JM (2010) ApuA, a multifunctional alpha-glucan-degrading enzyme of Streptococcus suis, mediates adhesion to porcine epithelium and mucus. Microbiology 156: 2818-2828.

33. de Greeff A, Buys H, Verhaar R, Dijkstra J, van Alphen L, et al. (2002) Contribution of fibronectin-binding protein to pathogenesis of Streptococcus suis serotype 2. Infect Immun 70: 1319-1325.

34. Esgleas M, Li Y, Hancock MA, Harel J, Dubreuil JD, et al. (2008) Isolation and characterization of alpha-enolase, a novel fibronectin-binding protein from Streptococcus suis. Microbiology 154: 2668-2679.

35. Jobin MC, Brassard J, Quessy S, Gottschalk M, Grenier D (2004) Acquisition of host plasmin activity by the Swine pathogen Streptococcus suis serotype 2. Infect Immun 72: 606-610.

36. Brassard J, Gottschalk M, Quessy S (2004) Cloning and purification of the Streptococcus suis serotype 2 glyceraldehyde-3-phosphate dehydrogenase and its involvement as an adhesin. Vet Microbiol 102: 87-94.

37. Tan C, Fu S, Liu M, Jin M, Liu J, et al. (2008) Cloning, expression and characterization of a cell wall surface protein, 6-phosphogluconate-dehydrogenase, of Streptococcus suis serotype 2. Vet Microbiol 130: 363-370.

38. Kouki A, Haataja S, Loimaranta V, Pulliainen AT, Nilsson UJ, et al. (2011) Identification of a novel streptococcal adhesin P (SadP) protein recognizing galactosyl-alpha1-4-galactose-containing glycoconjugates: convergent evolution of bacterial pathogens to binding of the same host receptor. J Biol Chem 286: 38854-38864.
